# Reducing Agent‐Mediated Nonenzymatic Conversion of 2‐Oxoglutarate to Succinate: Implications for Oxygenase Assays

**DOI:** 10.1002/cbic.202000185

**Published:** 2020-08-18

**Authors:** Amjad Khan, Christopher J. Schofield, Timothy D. W. Claridge

**Affiliations:** ^1^ Department of Chemistry University of Oxford 12 Mansfield Road Oxford OX1 3TA UK

**Keywords:** ascorbate, nonenzymatic, oxygenases, reducing agents, turnover

## Abstract

l‐Ascorbate (l‐Asc) is often added to assays with isolated Fe^II^‐ and 2‐oxoglutarate (2OG)‐dependent oxygenases to enhance activity. l‐Asc is proposed to be important in catalysis by some 2OG oxygenases *in vivo*. We report observations on the nonenzymatic conversion of 2OG to succinate, which is mediated by hydrogen peroxide generated by the reaction of l‐Asc and dioxygen. Slow nonenzymatic oxidation of 2OG to succinate occurs with some, but not all, other reducing agents commonly used in 2OG oxygenase assays. We intend these observations will help in the robust assignment of substrates and inhibitors for 2OG oxygenases.

## Graphical abstract

**Taking everything into account**: In catalytic reactions by some 2OG oxygenases with prolonged reaction times or high l‐ascorbate concentrations, nonenzymatic reactions of 2‐oxoacids mediated by reactive oxygen species such as H_2_O_2_ might be relevant. Knowledge of how catalysis is affected by, say, metal ions or temperature will allow appropriate controls to be implemented in kinetic studies or inhibition assays.

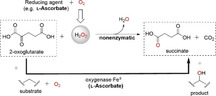

## Introduction

**Figure 1:**
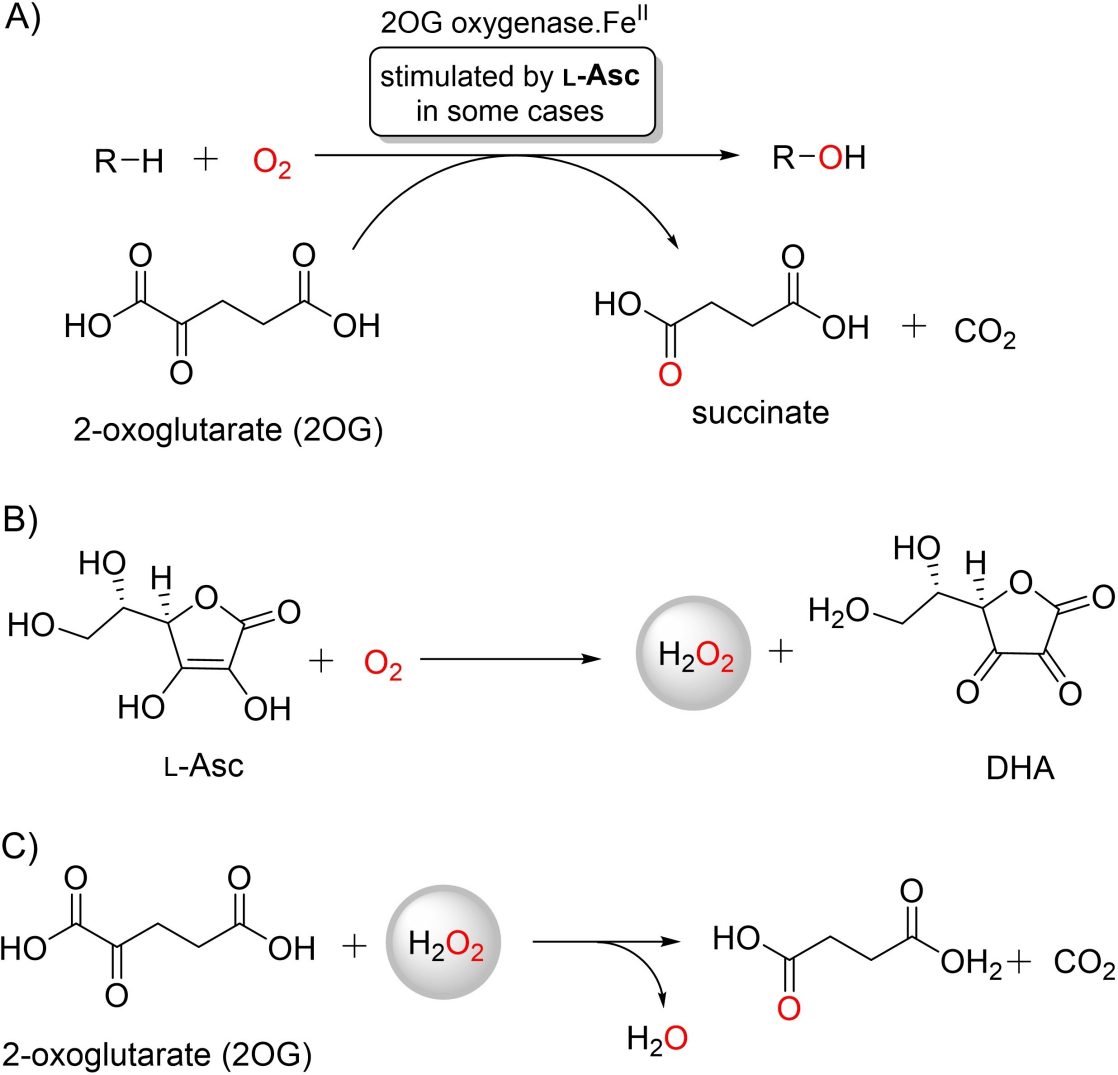
A 2OG oxygenase‐catalysed and H_2_O_2_‐mediated conversion of 2OG to succinate. A) Stoichiometry of a typical 2OG oxygenase‐catalysed hydroxylation. B) l‐Asc reacts with O_2_ to give H_2_O_2_ and dehydroascorbate (DHA). C) H_2_O_2_ reacts with 2OG to give succinate and CO_2_.

The Fe^II^‐ and 2‐oxoglutarate (2OG)‐dependent oxygenase superfamily of structurally and mechanistically related enzymes is widely distributed throughout much of biology.^1^, ^2^, ^3^, ^4^ Some human 2OG oxygenases are current therapeutic targets, with medicinal interest in them likely to increase with the recent clinical approval of inhibitors of the hypoxia‐inducible factor prolyl hydroxylases. Human 2OG oxygenases have roles in epigenetics, DNA/RNA damage repair/modification, lipid metabolism, hypoxia sensing, modification of the translational machinery, epidermal growth factor‐like protein modification, and in collagen biosynthesis.^1^, ^2^, ^3^, ^4^ Plant 2OG oxygenases are important agrochemical targets. 2OG oxygenase catalysis typically involves coupling of dioxygen‐mediated decarboxylation of 2OG with two‐electron substrate oxidation (commonly hydroxylation; Figure 1A). All identified 2OG oxygenases use Fe^II^ as a cofactor, though structurally homologous enzymes can bind other metals and catalyse other reaction types.^1^, ^2^, ^3^, ^4^

Pioneering work showed that l‐ascorbate (l‐Asc) enhances the activity of the procollagen prolyl‐hydroxylases.^5^ The role of l‐Asc in enhancing the activity of 2OG‐dependent procollagen prolyl hydroxylases is linked to the disease scurvy, which involves impaired collagen biosynthesis, due to lack of l‐Asc (and maybe other redox agents).^6^, ^7^, ^8^ The precise mechanism of the enhancement of the activity of some 2OG oxygenases by l‐Asc *in vivo* remains unknown.

The extent of the enhancement of isolated 2OG oxygenase activity by l‐Asc varies, for example, it is more pronounced for the procollagen prolyl‐4‐hydroxylases (P4H), where some studies report it to be a crucial requirement,^5^, ^9^, ^10^ than for some other enzymes. The activity of the ten‐eleven translocation (TET) 2OG oxygenases, which act on 5‐methyl cytosine nucleobases, is reported to be enhanced by the presence of l‐Asc in cells.^11^ The plant enzyme 1‐aminocyclopropane‐1‐carboxylic acid (ACC) oxidase is structurally related to 2OG oxygenases, but does not employ 2OG; it converts ACC into ethylene, and is reported to have a strong requirement for l‐Asc, at least in isolated form.^12^, ^13^

The processes by which l‐Asc enhances the activity of isolated 2OG oxygenases are potentially complex. No definitive l‐Asc binding site has been identified in any of the reported 2OG oxygenase X‐ray crystal structures.^14^ l‐Asc may promote 2OG oxygenase catalysis by one or more of the following or other related processes:^6^, ^9^, ^10^, ^15^, ^16^ i) by acting as a reducing agent to keep the Fe^II^ cofactor in its active Fe^II^ form, either in solution, or at the active site; ii) helping complete reaction cycles, where 2OG oxidation is uncoupled from that of the substrate; iii) maintaining cysteinyl thiols in the protein and/or substrate in the thiol form; iv) by directly promoting catalysis. There is thus clear potential for complexity in redox reactions including l‐Asc, O_2_, Fe^II^/Fe^III^, and protein/substrate thiols. l‐Asc is sometimes used in assays involving isolated enzymes from organisms that don't contain l‐Asc;^17^ hence, enhancement of isolated 2OG oxygenase activity by l‐Asc does not necessarily reflect biological relevance. Other reducing agents are sometimes used in 2OG oxygenase assays in addition to or instead of l‐Asc^18^ (see below).

Various assays are reported for 2OG oxygenases, both for use in functional assignment and inhibition studies.^2^, ^19^, ^20^, ^21^ Assays monitoring depletion of O_2_/2OG and/or production of succinate have been employed in substrate assignment studies/kinetic analyses and have frequently been used in inhibition studies.^2^, ^19^, ^20^, ^21^ The use of a dynamic redox mixture, in which l‐Asc is a common component, can complicate the interpretation of results.

**Figure 2:**
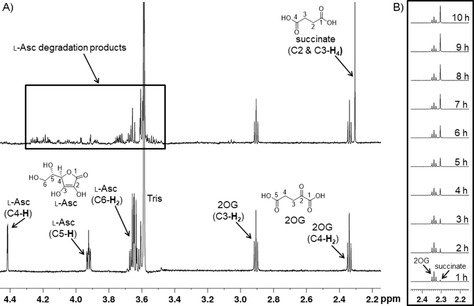
l‐Asc causes reaction of 2OG to give succinate under aerobic conditions. A) Overlay of ^1^H NMR spectra of a freshly prepared mixture of l‐Asc and 2OG in [D_11_]Tris buffer (bottom) compared with the same mixture after 10 hours (top). Concentrations used: 500 μM l‐Asc, 200 μM 2OG in aqueous 50 mM [D_11_]Tris, pH 7.5. After 10 hours, l‐Asc is completely reacted to give degradation products (signals in rectangle) with concomitant generation of succinate (88 μM). B) Time course of ^1^H NMR spectra (partial spectra shown for clarity) of l‐Asc/2OG/[D_11_]Tris buffer mixture for the shown times.

Herein, we report ^1^H NMR studies revealing that the amount of hydrogen peroxide (H_2_O_2_) generated by reaction of l‐Asc (and other reducing agents used in 2OG oxygenase assays) with dioxygen can be sufficient to enable the nonenzymatic conversion of 2OG to succinate (Figures 1B, C and 2). We suggest that the observations should be considered in 2OG oxygenase assays employing l‐Asc or other reducing agents, especially under conditions where enzyme activity may be attenuated, such as in the presence of an inhibitor or by modification of wild‐type enzyme.

## Results and Discussion

**Figure 3:**
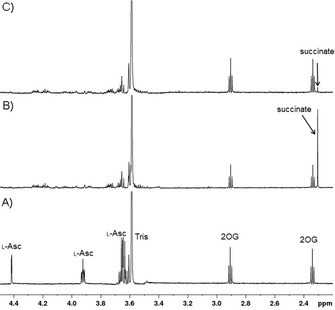
The effect of Fe^II^ on the l‐Asc‐mediated reaction of 2OG to succinate. ^1^H NMR spectra of A) a freshly prepared mixture of l‐Asc/2OG/[D_11_]Tris buffer, B) the same mixture after 10 hours’ incubation without Fe^II^ or C) after 10 hours with Fe^II^. Note that in the presence of Fe^II^, reduced conversion of 2OG to succinate is observed. Concentrations used: 500 μM l‐Asc, 200 μM 2OG, 100 μM Fe^II^ in aqueous 50 mM [D_11_]Tris, pH 7.5.

When l‐Asc is incubated with 2OG in the absence of an enzyme in aqueous [D_11_]Tris buffer at room temperature, a substantial conversion of 2OG to succinate is observed (Figures 2 and S1). Time course analysis reveals that l‐Asc reacts slowly to give degradation products including dehydroascorbate (DHA)^22^ with concomitant conversion of 2OG to succinate (Figures 2B and S2). Substitution of l‐Asc with DHA also results in the conversion of 2OG to succinate, but to a lower extent compared to l‐Asc (Figure S3). The production of succinate from 2OG when using l‐Asc is ablated by addition of catalase (which catalyses the decomposition of H_2_O_2_ to give H_2_O and O_2_;^23^ Figure S4), an observation consistent with the H_2_O_2_‐mediated conversion of 2OG to succinate (Figure S5).The results also indicate that catalase inhibits degradation of l‐Asc in the presence of dioxygen, consistent with the reported degradation of l‐Asc by H_2_O_2_.^24^ Incubation of l‐Asc under anaerobic conditions did not manifest any 2OG conversion to succinate (Figure S6), consistent with the reaction of l‐Asc (and DHA) and dioxygen to give H_2_O_2_, as reported.^25^, ^26^, ^27^, ^28^ Addition of Fe^II^ to the l‐Asc/2OG/buffer mixture inhibited the reaction of 2OG (Figure 3). This observation likely reflects oxidation of Fe^II^ by H_2_O_2_ so reducing the amount of H_2_O_2_ available to oxidise 2OG (Figure S7).

Studies with other metal ions commonly used in 2OG oxygenase assay mixtures reveal that Na^I^, K^I^, Mg^II^, Ca^II^ and Ni^II^ ions have no detectable effect on the l‐Asc‐mediated 2OG reaction to succinate (Figure S8). Cu^II^ ions manifest an inhibitory effect (similar to Fe^II^; Figure S8), possibly due to reaction with H_2_O_2_. Zn^II^ ions manifested a mild stimulatory effect (Figures S8 and S9), possibly involving complexation with 2OG and/or l‐Asc.

Studies with buffers commonly used in 2OG oxygenase assays reveal differences in the rates of l‐Asc‐mediated 2OG to succinate conversion (Figure S10). Thus, [D_11_]Tris, HEPES, PIPES and MOPS appeared to promote the reaction better than sodium phosphate, tricine or TES buffers (Figure S10).

**Figure 4:**
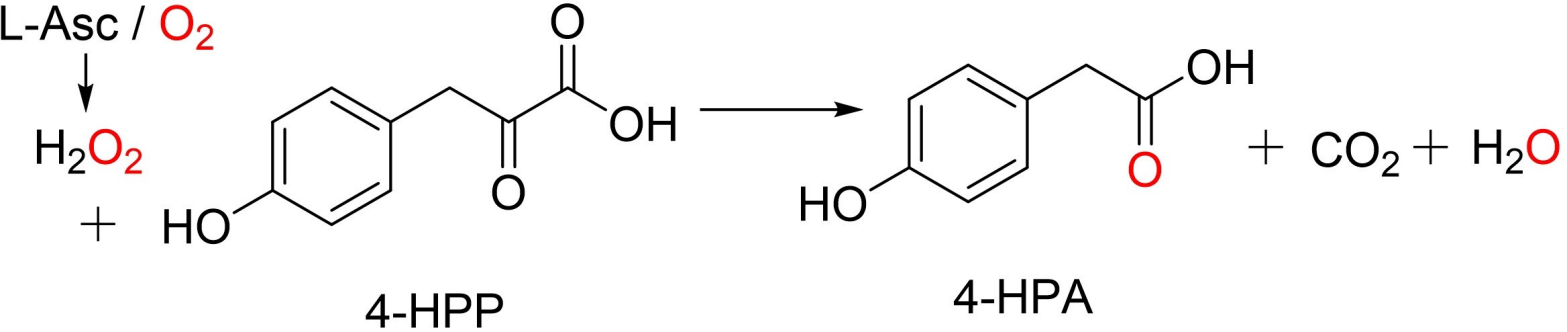
l‐Asc‐mediated reaction of 4‐hydroxyphenyl pyruvate (4‐HPP) to 4‐hydroxyphenyl acetate (4‐HPA). l‐Asc and dioxygen reacts and generates hydrogen peroxide, which reacts with 4‐HPP to give 4‐HPA.

To investigate whether l‐Asc‐mediated oxidative decarboxylation extends to other 2‐oxoacids, we studied reactions using 4‐hydroxyphenylpyruvate (4‐HPP; Figures 4 and S11), pyruvate (Figure S12) and oxaloacetate salts (Figure S13). All the 2‐oxoacids underwent l‐Asc‐mediated decarboxylation. In the case of oxaloacetate, we observed oxidative decarboxylation to give malonate, which apparently underwent further (nonoxidative) decarboxylation to give acetate. With pyruvate, Fe^II^ and catalase displayed a similar inhibitory effect on oxidative decarboxylation to that observed with 2OG (Figure S14). Citrate (Figure S15), dl‐isocitrate (Figure S16), malate (Figure S17) and 1,3 dicarbonyl compounds including, benzoylacetone, methylacetoacetate and 2,4 pentanedione, were not observed to undergo reaction despite the oxidation of l‐Asc.

**Figure 5:**
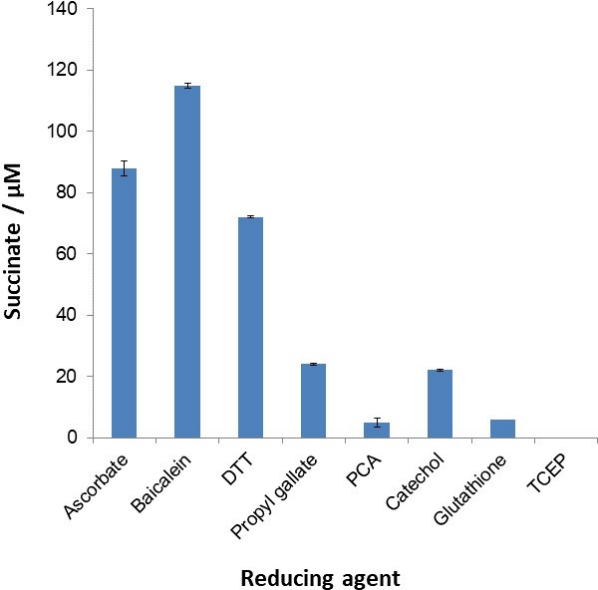
2OG conversion to succinate with different reducing agents. The extent of 2OG conversion to succinate in the presence of different reducing agents: l‐Asc, baicalein, DTT, propyl gallate, PCA, catechol, glutathione and TCEP. Concentrations used: 500 μM reducing agent, 200 μM 2OG in aqueous 50 mM [D_11_]Tris, pH 7.5. Error bars represent standard deviations from the mean (*n*=3) of three separate measurements.

Other potential biological reducing agents/l‐Asc analogues (Figure 5) including dithiothreitol (DTT; Figure S18), the flavone baicalein (Figure S19), propyl gallate (Figure S20), protocatechuic acid (PCA; Figure S21), catechol (Figure S22), glutathione (GSH; Figure S23) and tris(2‐carboxyethyl)phosphine hydrochloride (TCEP; Figure S24) were tested for their ability to promote the reaction of 2OG to succinate. The results reveal that most of these reducing agents promote the reaction of 2OG to give succinate, though to different extents (Figure 5). The levels of 2OG turnover appear to approximately correlate with the extent of redox agent oxidation. Baicalein and DTT promoted the reaction to a similar level to that observed for l‐Asc, while, relatively low levels of 2OG turnover were observed with propyl gallate, PCA, catechol, or glutathione. Under the standard tested conditions, TCEP did not promote the turnover of 2OG to give succinate.

## Conclusions

The overall results indicate that the reaction of 2OG to give succinate under conditions often used for assays of 2OG oxygenases can be mediated by H_2_O_2_,^29^, ^30^, ^31^, ^32^, ^33^, ^34^ produced by the reaction of l‐Asc and dioxygen (Figures 1 and 2).^25^, ^27^ Analogous results were obtained for other 2‐oxoacids, including 4‐hydroxyphenylpyruvate, which is the substrate for *p*‐hydroxyphenylpyruvate dioxygenase. Given the slow nature of the reducing agent‐catalysed nonenzyme 2OG conversion to succinate (0.13 μM min^−1^ for l‐Asc), at least under our experimental conditions, it is possible that such nonenzymatic reactions of 2OG/other 2‐oxoacids may be particularly relevant when prolonged reaction times and/or high l‐Asc concentrations are used. It may also be important for inhibition studies or studies with substituted proteins where the normal enzyme activity is attenuated. Reactions between dioxygen and other redox active agents used in 2OG oxygenase‐catalysed reactions, such as DTT, are also likely to produce H_2_O_2_. Under our tested conditions it is notable that TCEP did not promote the conversion of 2OG to succinate thus, at least for some 2OG oxygenase assays, TCEP might be a preferred reducing agent. However, the ability of TCEP to coordinate metals ions requires consideration.^35^ It is possible that redox‐reactive intermediates produced at 2OG oxygenase active sites (either in catalytically active or inactive forms) may produce reactive oxygen species (ROS), including H_2_O_2_, that mediate the nonenzymatic conversion of 2OG to succinate. H_2_O_2_ (or other ROS) may also inhibit catalysis by oxidising Fe^II^, either in bulk solvent or at the active site. The extent of these processes will be affected by the exact nature of the particular 2OG oxygenase, including how tightly the Fe^II^ ion is bound at the active site. Fe^II^ is required by 2OG oxygenases for catalysis, but as shown here, can inhibit conversion of 2OG to succinate in the presence of l‐Asc. Moreover, high‐valent enzyme–Fe complexes in 2OG oxygenase catalysis can undergo inactivation^36^ and l‐Asc/DHA undergo oxidative degradation.^22^, ^24^, ^26^, ^27^ Other variables include the rate of H_2_O_2_ consumption, the presence of other metal ions, the buffer used, and the temperature. In the latter regard it is notable that O_2_ solubility in aqueous media decreases with increases in temperature.^37^ The nature of the substrate/product might also be a factor, because substrate oxidation can be uncoupled from that of 2OG, and the residence time of the product on the enzyme may affect active site Fe accessibility to ROS.

The results presented here, and previously,^22^, ^23^, ^25^, ^27^, ^29^, ^30^, ^31^, ^32^, ^33^ thus suggest that if 2OG/O_2_ consumption or succinate/CO_2_ production are being used for kinetic studies or inhibition assays, appropriate controls should be implemented. The different extents to which different redox agents promote the nonenzymatic reaction of 2OG should be considered. Our view is that the direct observation of substrate depletion or product formation (e. g., as measured by MS or NMR) are preferred methods for 2OG oxygenase assays.^2^, ^38^ However, although cosubstrate/coproduct measurements are not ideal for assignment of substrates/products, they can have utility in efficient/cost effective inhibition assays, though appropriate controls should be used.

Finally, given the high levels of l‐Asc (and other redox agents, e. g., transition metal ions) commonly used in cellular studies, we suggest that consideration should be given to possible nonenzymatic reactions involving redox labile metabolites, such as l‐Asc and 2OG in cells. The potential of such reactions is highlighted by recent work on the possible role of Fe in the evolution of the tricarboxylic acid cycle.^39^

## Experimental Section

**Materials**: Reagents were from Acros, Alfa Aesar, Apollo Scientific, Cambridge Isotope Laboratories, Sigma‐Aldrich or Cortecnet.

^**1**^ **H NMR experiments**: Analyses were carried out by recording ^1^H NMR spectra at 298 K using a Bruker Avance III 700 MHz spectrometer equipped with an inverse TCI cryoprobe, in 3 mm diameter Bruker MATCH NMR tubes with a total sample volume of 160 μL. All assay mixtures (total volume 144 μL) were prepared in 50 mM [D_11_]Tris (pH 7.5) buffer dissolved in H_2_O and incubated in 1.5 mL transparent Eppendorf tubes at room temperature. At the end of the incubation, 10 % D_2_O (16 μL) was added to these samples as a spectrometer deuterium lock signal. Spectra were recorded within 5 min after the incubation times. Each ^1^H NMR experiment was recorded with three separately prepared samples. Spectra were processed using TopSpin 3.1 software with a Lorentzian line broadening of 0.3 Hz. Water suppression was achieved by excitation sculpting suppression. Stock solutions of l‐Asc (monosodium salt, 25 mM) and 2OG (monosodium salt, 16 mM) were freshly prepared in Milli‐Q H_2_O. Catalase (bovine liver) was added as an aqueous suspension (3 μL, 45 mg/mL, 1735 units). Sodium, potassium, magnesium, calcium, zinc, copper, nickel, manganese, and cobalt stock solutions were made from the corresponding chloride salts (NaCl, KCl, MgCl_2_, CaCl_2_, ZnCl_2_, CuCl_2_, NiCl_2_). The Fe^II^ stock solution (250 mM) was freshly prepared using (NH_4_)_2_Fe(SO_4_)_2_ **⋅**6H_2_O in 20 mM HCl in H_2_O, which was then diluted to appropriate concentration.

## Conflict of interest

The authors declare no conflict of interest.

## Supporting information

As a service to our authors and readers, this journal provides supporting information supplied by the authors. Such materials are peer reviewed and may be re‐organized for online delivery, but are not copy‐edited or typeset. Technical support issues arising from supporting information (other than missing files) should be addressed to the authors.

Supplementary
