# Supplementary material for: Reducing Agent‐Mediated Nonenzymatic Conversion of 2‐Oxoglutarate to Succinate: Implications for Oxygenase Assays
Source: Chembiochem. 2020 Aug 18;21(20):2898–902. doi: 10.1002/cbic.202000185 (PMC7693218; doi:10.1002/cbic.202000185)
Supplement: Supplementary file 1 — Supplementary [file CBIC-21-2898-s001.pdf]

ChemBioChem

Supporting Information

Reducing Agent-Mediated Nonenzymatic Conversion of 2-Oxoglutarate to Succinate: Implications for Oxygenase Assays

Amjad Khan, Christopher J. Schofield,* and Timothy D. W. Claridge*

Table of contents

Figure S1. Stabilities of 2OG and L-Asc in Tris-D_{11} buffer. 3

Figure S2. ^{1}H NMR time course analysis of L-Asc mediated 2OG conversion to succinate. 4

Figure S3. Dehydroascorbate (DHA) mediated conversion of 2OG to succinate. 5

Figure S4. The effect of catalase on L-Asc mediated conversion of 2OG to succinate. 6

Figure S5. H_2O_2-mediated 2OG conversion to succinate. 7

Figure S6. The effect of oxygen on the L-Asc/2OG/Tris-D_{11} buffer incubation assay. 8

Figure S7. The effect of Fe(II) on H_2O_2-mediated 2OG conversion to succinate. 9

Figure S8. The effect of different metal ions on the L-Asc mediated 2OG conversion to succinate. 10

Figure S9. The effect of Zn(II) on L-Asc mediated 2OG conversion to succinate. 11

Figure S10. The effect of different buffers on L-Asc mediated 2OG conversion to succinate. 12

Figure S11. L-Asc mediated reaction of 4-hydroxyphenyl pyruvate (4-HPP) to give 4-hydroxyphenyl acetic acid (4-HPA). 14

Figure S12. L-Asc-mediated conversion of pyruvate to acetate. 16

Figure S13. L-Asc mediated conversion of oxaloacetate to malonate and acetate. 18

Figure S14. The effects of Fe(II), Zn(II) and catalase on the L-Asc mediated conversion of pyruvate to acetate. 19

Figure S15. L-Asc and citrate incubation assay. 20

Figure S16. L-Asc and DL-isocitrate incubation assay. 21

Figure S17. L-Asc and malate incubation assay. 22

Figure S18. Dithiothreitol (DTT) – mediated 2OG conversion to succinate. 23

Figure S19. Baicalein-mediated 2OG conversion to succinate. 24

Figure S20. Propyl gallate-mediated conversion of 2OG to succinate. 25

Figure S21. Protocatechuic acid (PCA)-mediated 2OG conversion to succinate. 26

Figure S22. Catechol-mediated 2OG reaction to succinate. 27

Figure S23. Glutathione (GSH)-mediated 2OG conversion into succinate. 28

Figure S24. Tris(2-carboxyethyl)phosphine (TCEP) – 2OG incubation assay. 29

(a)

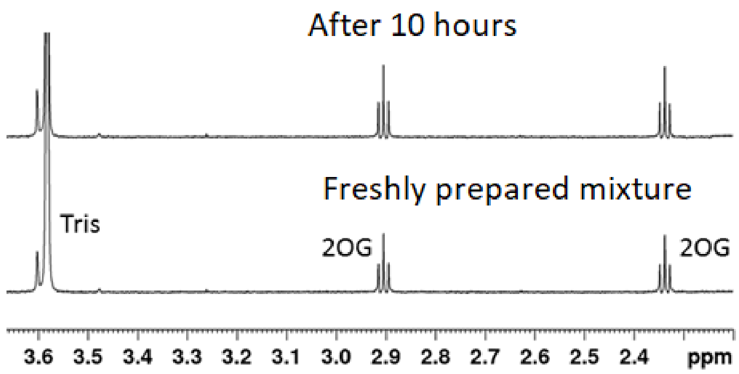

(b)

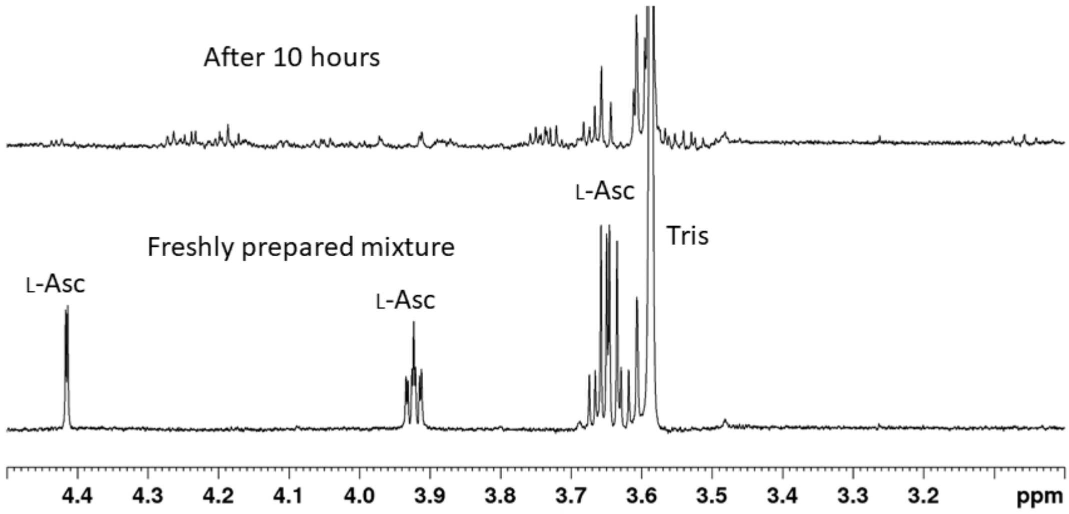

Figure S1. Stabilities of 2OG and L-Asc in Tris-D_{11} buffer.

(a) Overlay of ^{1}H NMR spectra of a freshly prepared mixture of 2OG in aqueous Tris-D_{11} buffer (bottom) compared with the same mixture after 10 hours (top). (b) Overlay of ^{1}H NMR spectra of a freshly prepared mixture of L-Asc in aqueous Tris-D_{11} buffer (bottom) compared with the same mixture after 10 hours (top). Concentrations used: 2OG 200 µM, L-Asc 500 µM in 50 mM aqueous Tris-D_{11} at pH 7.5.

(a)

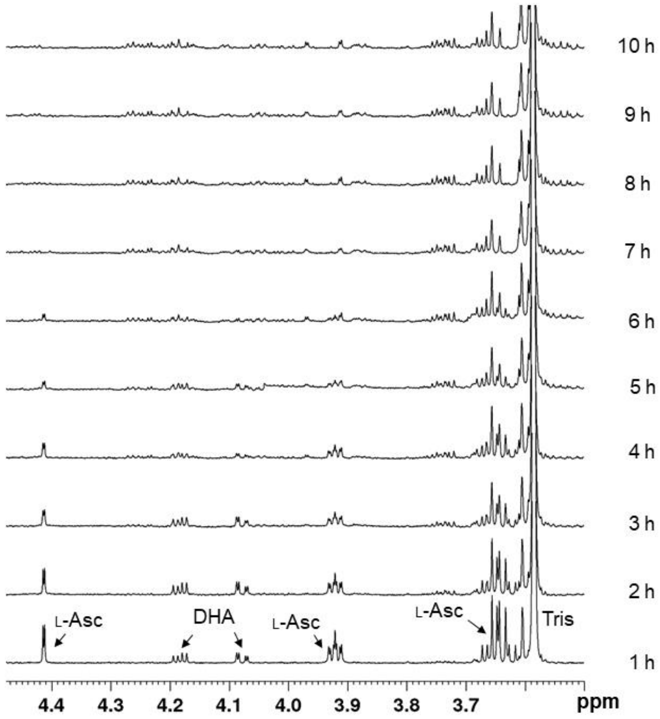

(b)

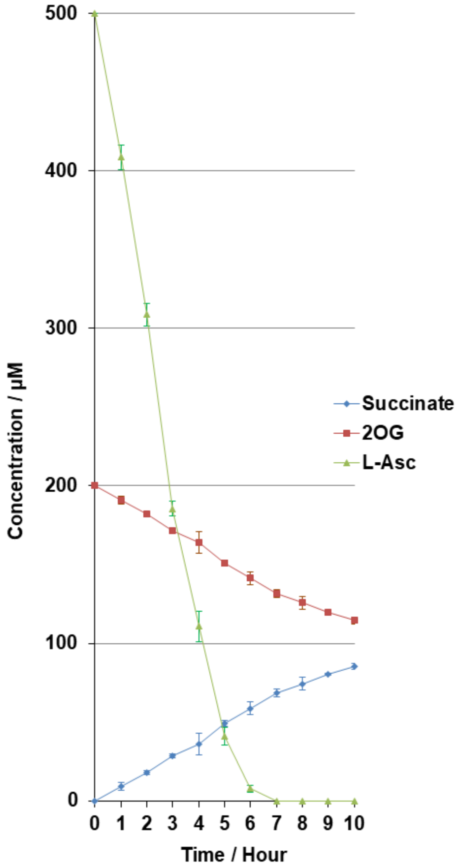

Figure S2. ^{1}H NMR time course analysis of L-Asc mediated 2OG conversion to succinate.
(a) Overlay of ^{1}H NMR spectra (partial spectra are shown for clarity) of an L-Asc/2OG/Tris-D_{11} buffer mixture for the shown times. (b) Plot showing L-Asc degradation and simultaneous 2OG reaction to give succinate over time. Concentrations used: 500 µM L-Asc, 200 µM 2OG in 50 mM aqueous Tris-D_{11} at pH 7.5. DHA: Dehydroascorbate. Error bars represent standard deviations from the mean (n =3) of three separate measurements.

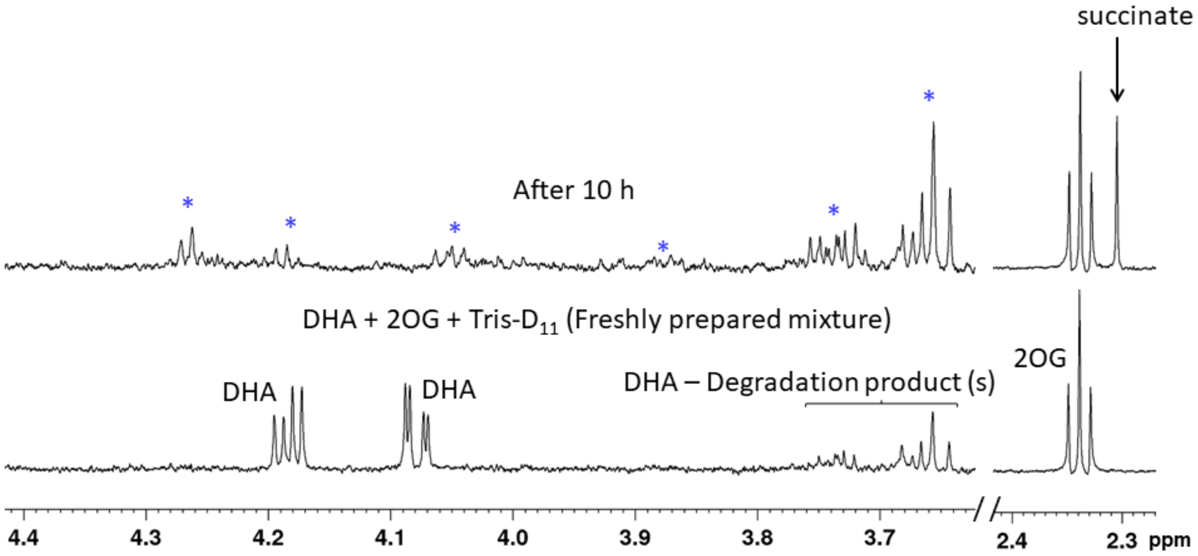

Figure S3. Dehydroascorbate (DHA) mediated conversion of 2OG to succinate.
Overlay of ^{1}H NMR spectra (partial spectra are shown for clarity) of a freshly prepared mixture of dehydroascorbate (DHA) and 2OG in aqueous Tris-D_{11} buffer (bottom) compared with the same mixture after 10 hours (top). Signals marked with asterisks are likely due to DHA degradation products. Concentrations used: 500 µM DHA, 200 µM 2OG in 50 mM aqueous Tris-D_{11}, pH 7.5.

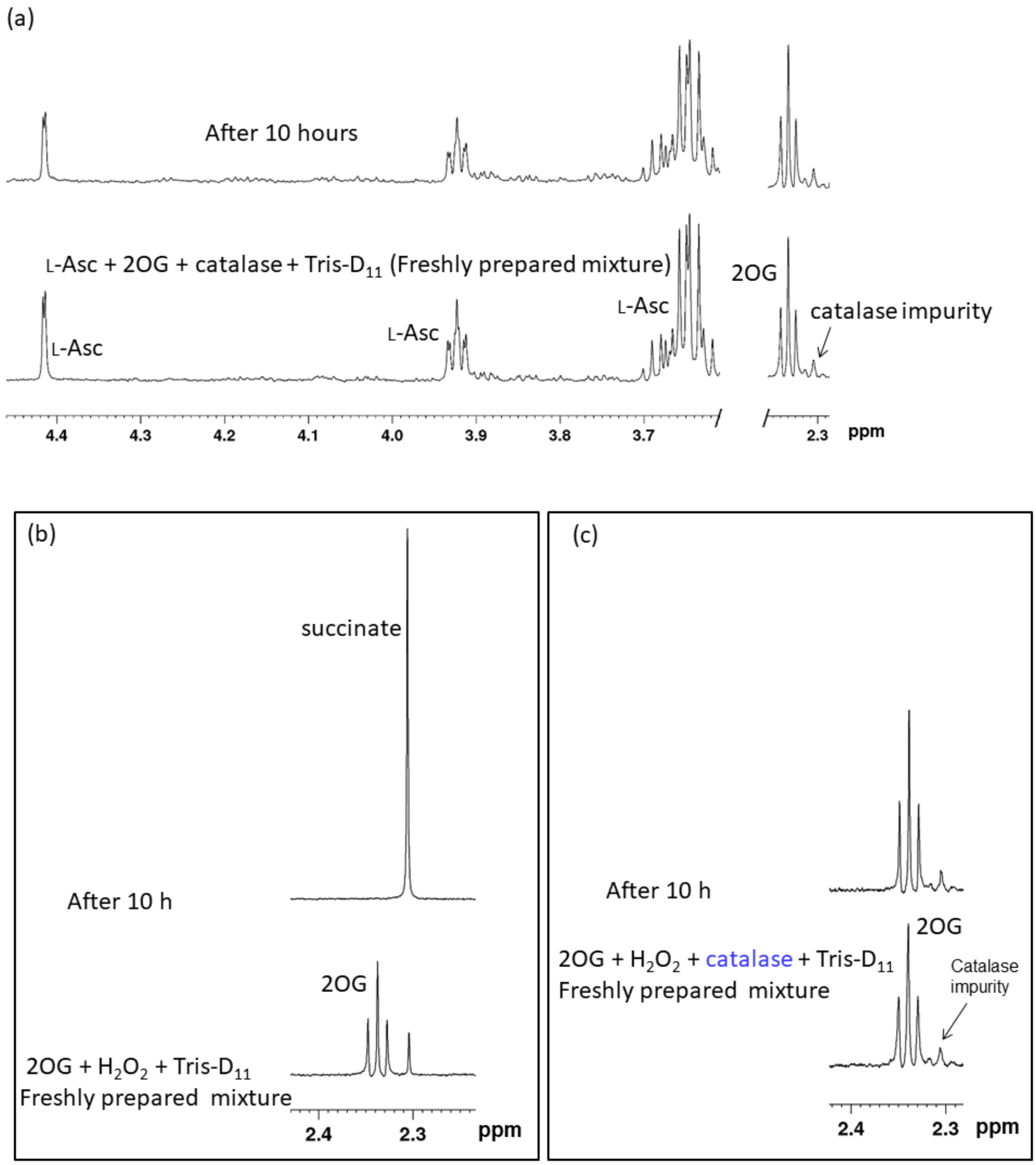

Figure S4. The effect of catalase on L-Asc mediated conversion of 2OG to succinate.

(a) Overlay of ^1H NMR spectra (partial spectra are shown for clarity) of a freshly prepared mixture of L-Asc/2OG/catalase/Tris-D_{11} buffer (bottom) compared with the same mixture after 10 hours (top). In the presence of catalase, no 2OG conversion to succinate is observed. (b) Overlay of ^1H NMR spectra (partial spectra are shown for clarity) of a freshly prepared mixture of 2OG/H_2O_2/Tris-D_{11} buffer (bottom) compared with the same mixture after 10 hours (top). (c) Overlay of ^1H NMR spectra (partial spectra are shown for clarity) of a freshly prepared mixture of 2OG/H_2O_2/catalase/Tris-D_{11} buffer (bottom) compared with the same mixture after 10 hours (top). In the presence of catalase, H_2O_2 mediated 2OG conversion to succinate is not observed. Concentrations used: 500 µM L-Asc, 200 µM 2OG, 500 µM H_2O_2, 1735 units catalase in 50 mM aqueous Tris-D_{11} at pH 7.5.

(a)

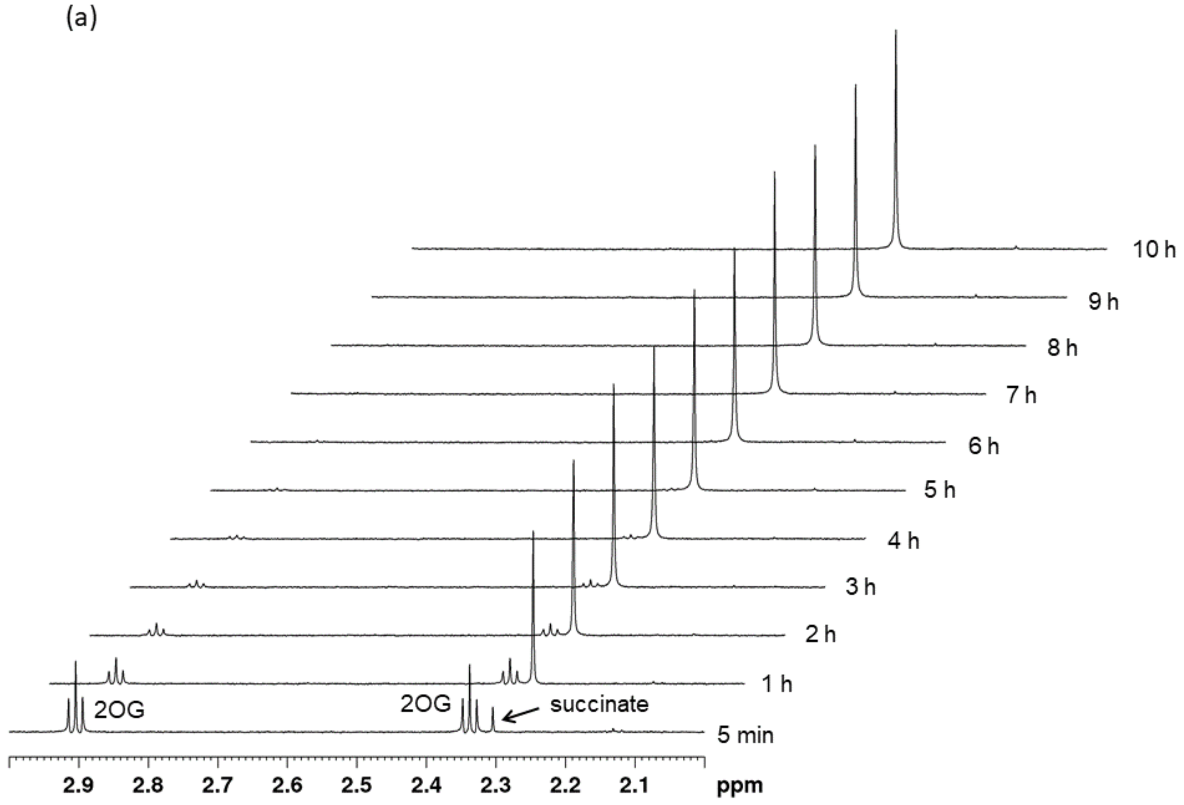

(b)

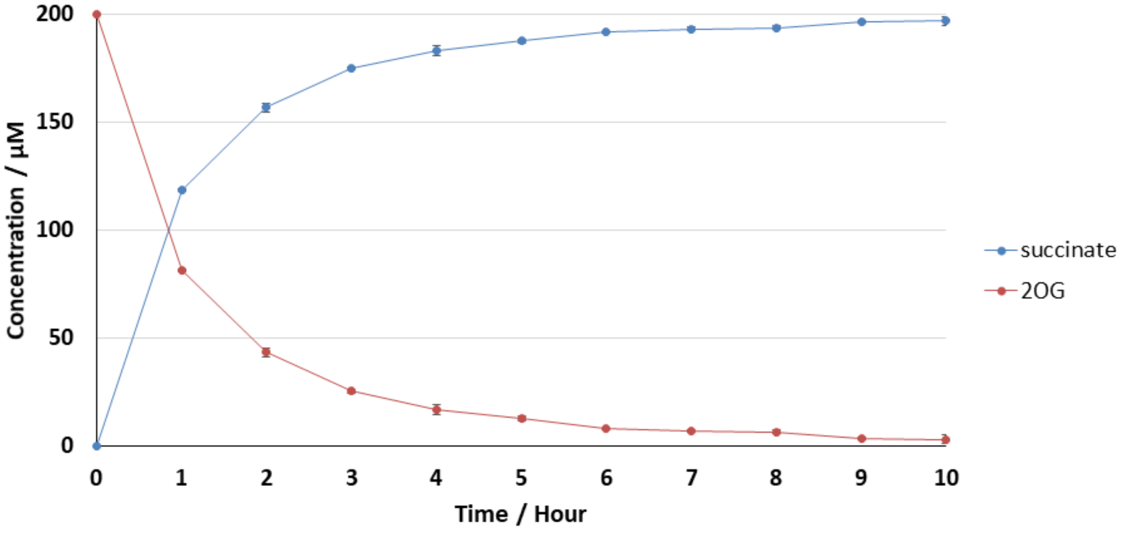

Figure S5. H_2O_2-mediated 2OG conversion to succinate.

(a) ^{1}H NMR time course analysis of the reaction of 2OG with H_2O_2 to form succinate. (b) Plot of 2OG conversion to succinate vs time (1-10 hours). Concentrations used: 500 μM H_2O_2, 200 μM 2OG in 50 mM aqueous Tris-D_{11} at pH 7.5. Error bars represent standard deviations from the mean (n =3) of three separate measurements.

(a)

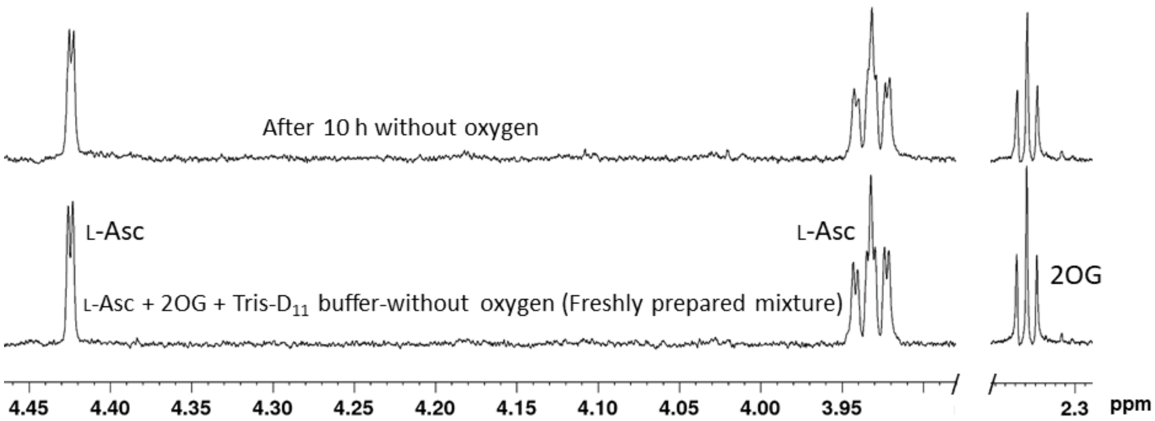

(b)

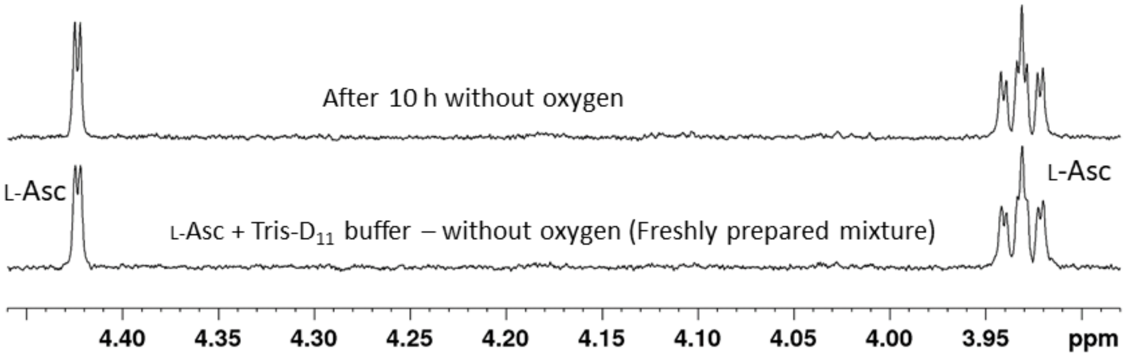

Figure S6. The effect of oxygen on the L-Asc/2OG/Tris-D_{11} buffer incubation assay.

(a) Overlay of ^{1}H NMR spectra (partial spectra are shown for clarity) of a freshly prepared mixture of L-Asc/2OG/Tris-D_{11} buffer under anaerobic conditions (bottom) compared with the same mixture after 10 hours (top). (b) Overlay of ^{1}H NMR spectra (partial spectra are shown for clarity) of a freshly prepared mixture of L-Asc in Tris-D_{11} buffer under anaerobic conditions (bottom) compared with the same mixture after 10 hours (top). These results imply that under anaerobic conditions, L-Asc does not undergo oxidation, hence, does not generate H_2O_2, and no conversion of 2OG to succinate occurs. Concentrations used: 500 µM L-Asc, 200 µM 2OG in 50 mM aqueous Tris-D_{11} at pH 7.5.

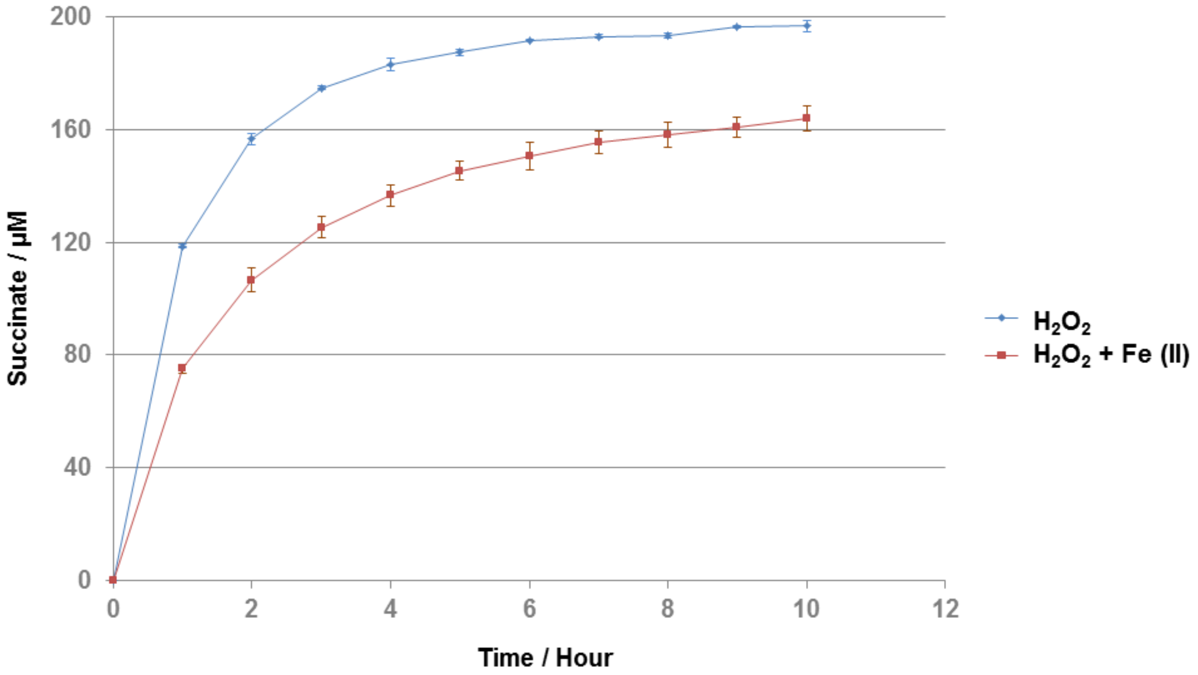

Figure S7. The effect of Fe(II) on H_2O_2-mediated 2OG conversion to succinate.

(a) ^1H NMR time course analysis of H_2O_2-mediated conversion of 2OG to succinate in the absence (blue curve) and presence (brown curve) of Fe(II). Concentrations used: 200 μM 2OG, 500 μM H_2O_2, 100 μM Fe(II) in 50 mM aqueous Tris-D_{11} at pH 7.5. Error bars represent standard deviations from the mean (n =3) of three separate measurements.

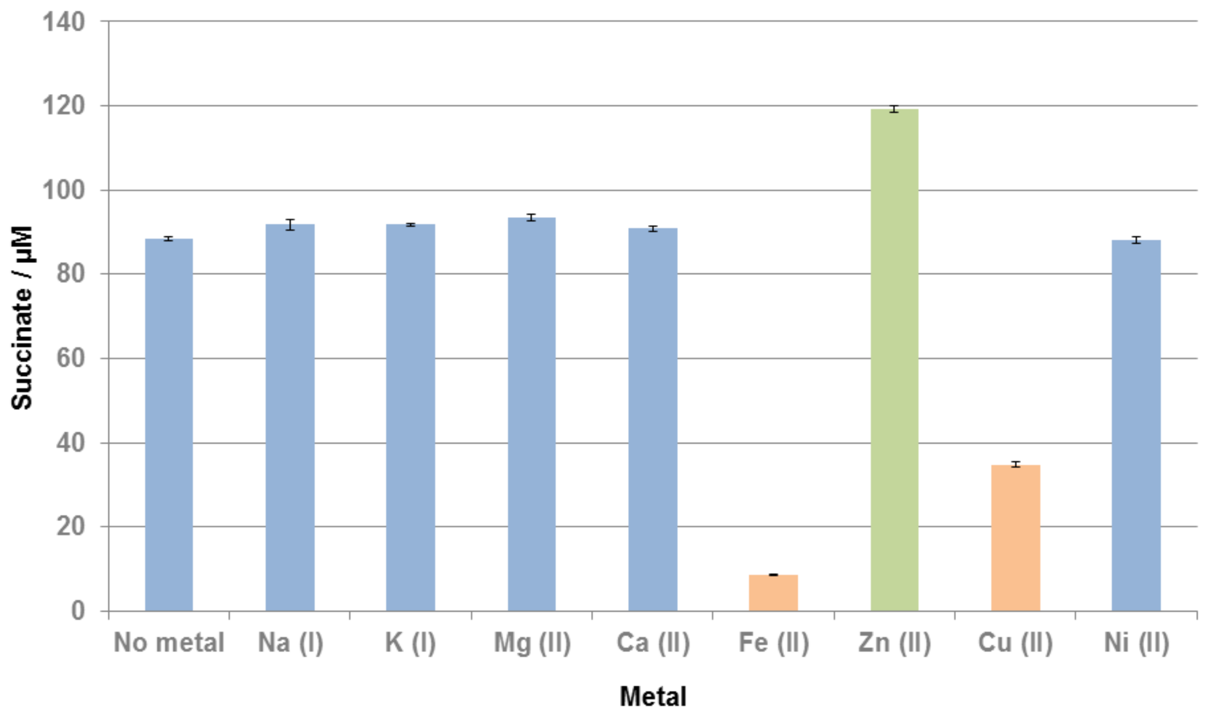

Figure S8. The effect of different metal ions on the L-Asc mediated 2OG conversion to succinate.

Chart showing the extent of 2OG conversion to succinate on incubating a mixture of L-Asc/2OG/metal ion/aqueous Tris-D_{11} buffer for 10 hours. Concentrations used: 500 µM L-Asc, 200 µM 2OG, 100 µM metal ion in 50 mM aqueous Tris-D_{11} buffer at pH 7.5. Error bars represent standard deviations from the mean (n = 3) of three separate measurements.

(a)

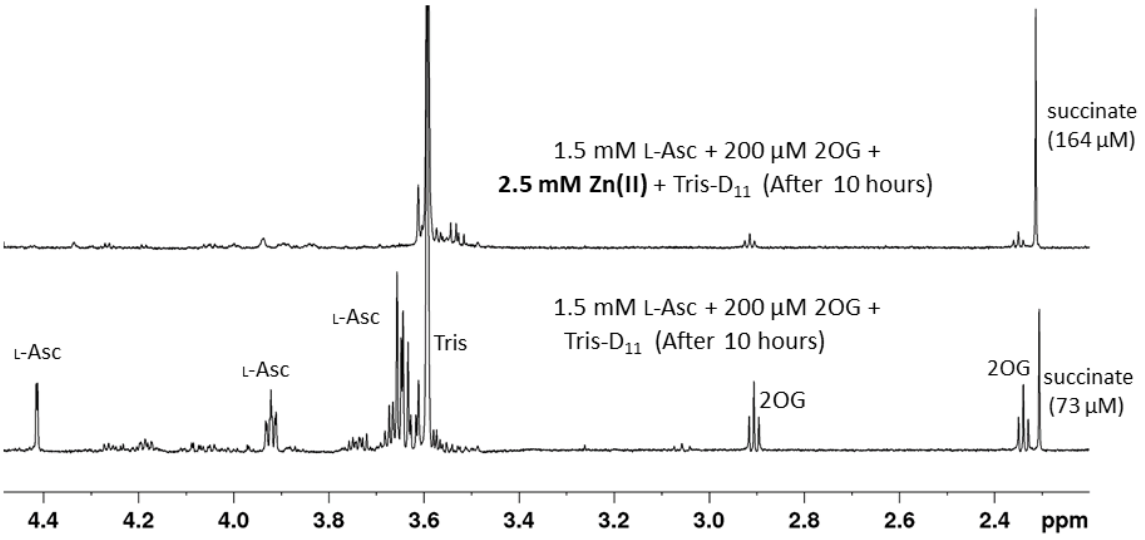

(b)

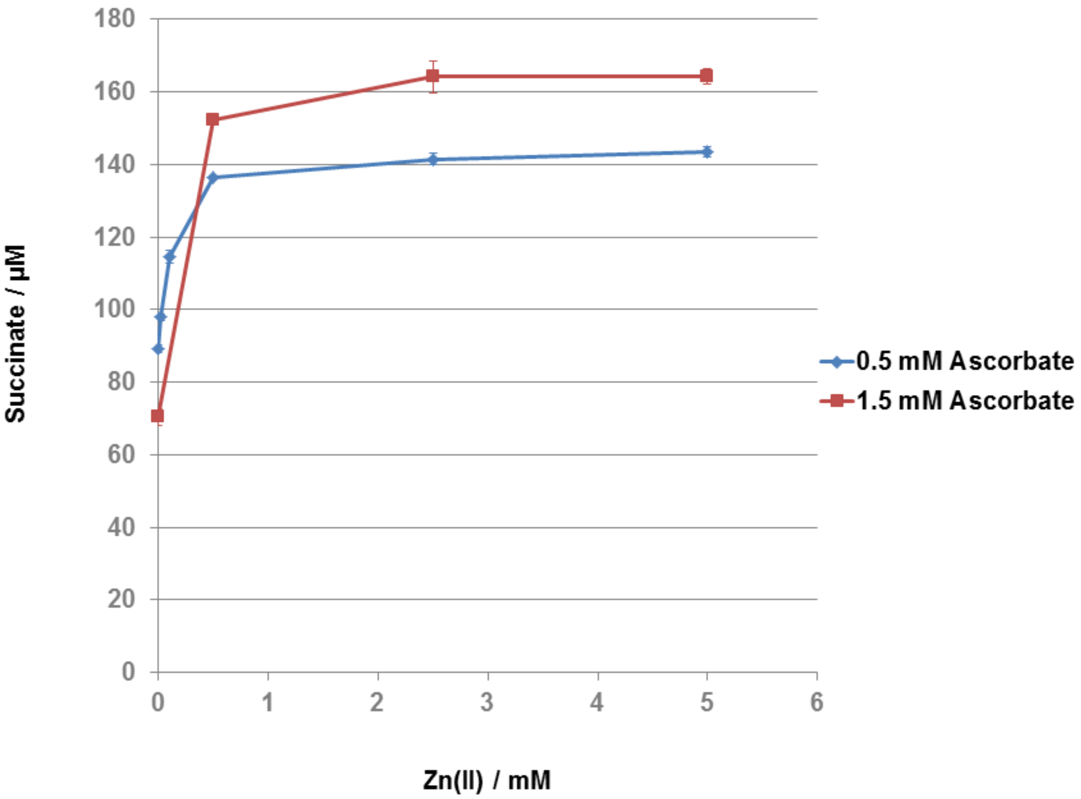

Figure S9. The effect of Zn(II) on L-Asc mediated 2OG conversion to succinate.

(a) Overlay of ^{1}H NMR spectra of a 10 hour incubation mixture of L-Asc/2OG/aqueous Tris-D_{11} buffer without Zn(II) (bottom) compared with the same mixture with Zn(II) (top). In the presence of Zn(II), L-Asc undergoes efficient oxidation enabling succinate formation. (b) The effect of different Zn(II) concentrations on the reaction of L-Asc . Concentrations used: Zn(II); 25 µM, 100 µM, 500 µM, 2.5 mM, 5 mM, L-Asc; 500 µM & 1.5 mM, and 2OG 200 µM in 50 mM aqueous Tris-D_{11} buffer at pH 7.5. Error bars represent standard deviations from the mean (n = 3) of three separate measurements.

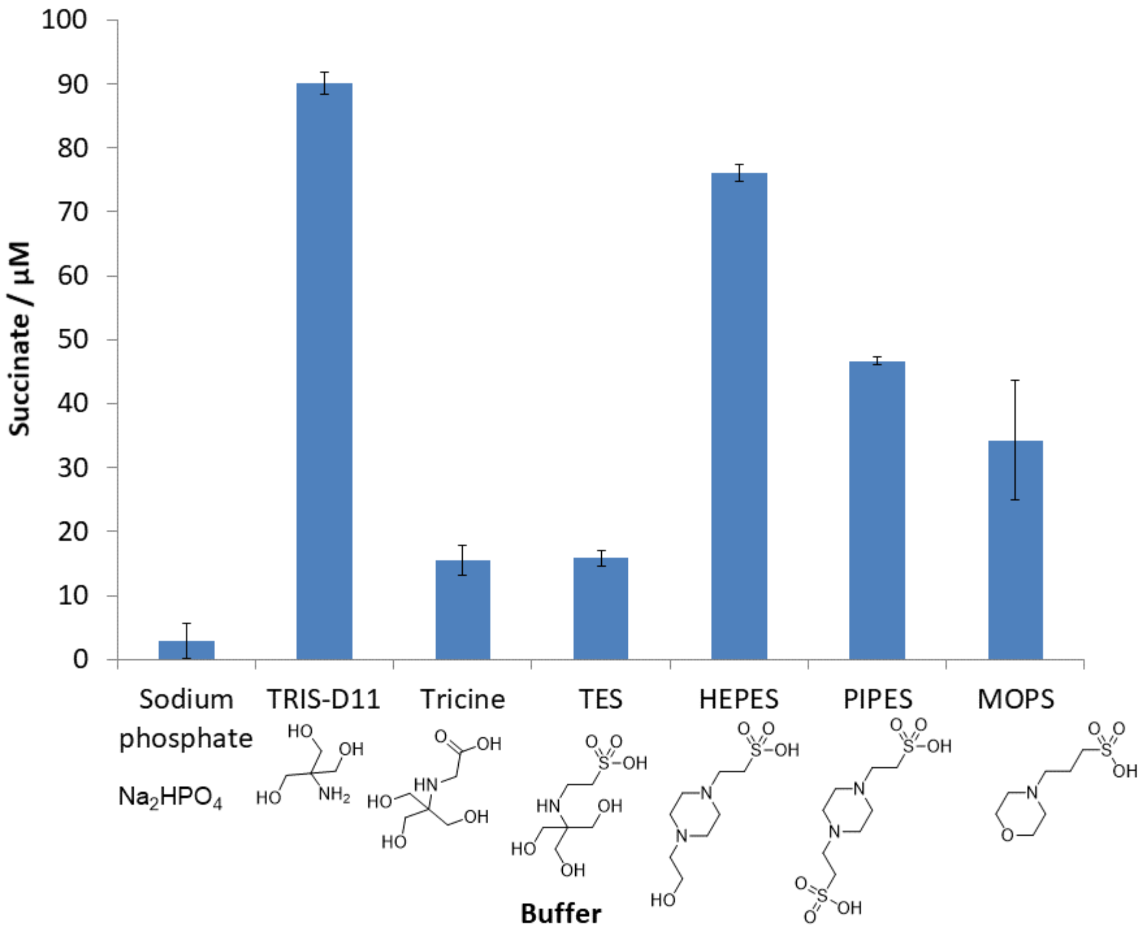

Figure S10. The effect of different buffers on L-Asc mediated 2OG conversion to succinate.
Chart showing the extent of 2OG conversion to succinate on incubating a mixture of L-Asc/2OG/aqueous buffer for 10 hours. Buffers used: sodium phosphate, Tris-D_{11}, tricine, TES, HEPES, PIPES and MOPS. Concentrations used: 500 µM L-Asc, 200 µM 2OG in 50 mM aqueous buffer at pH 7.5. Error bars represent standard deviations from the mean (n = 3) of three separate measurements.

(a)

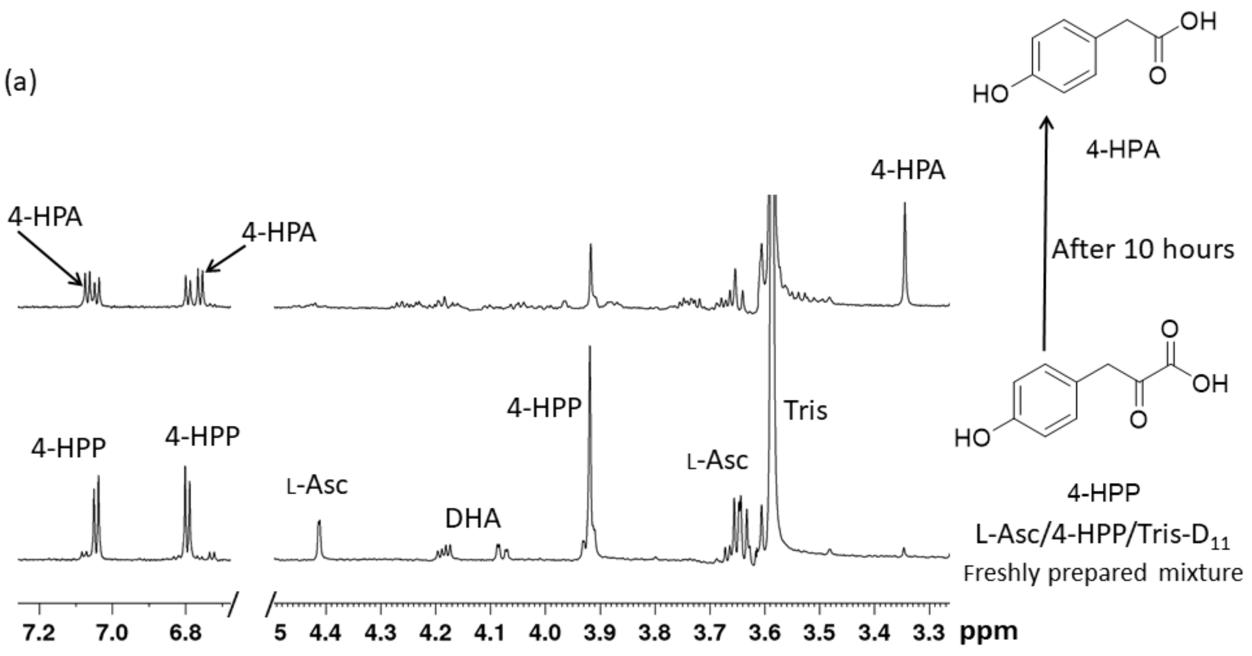

(b)

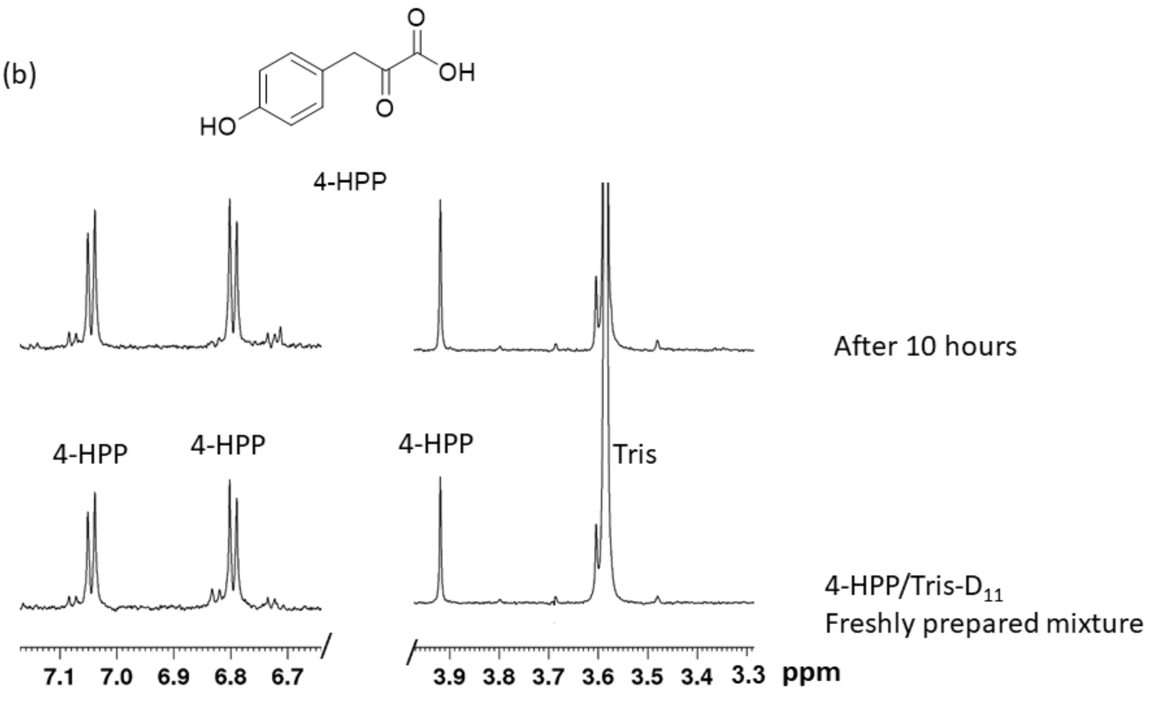

(Figure continues)

(C)

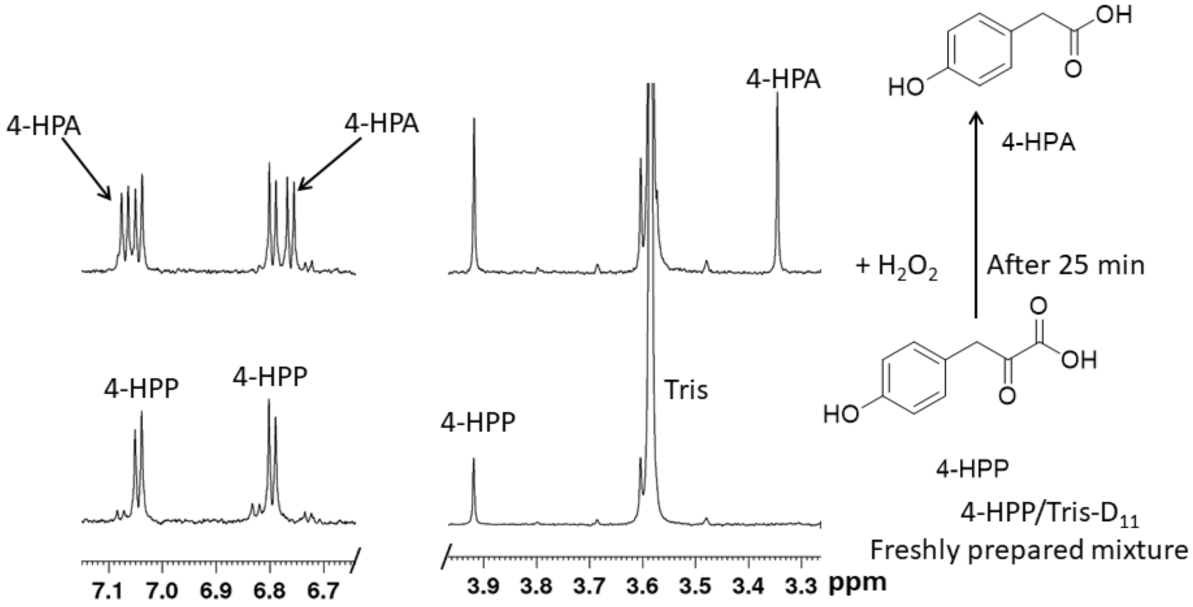

Figure S11. L-Asc mediated reaction of 4-hydroxyphenyl pyruvate (4-HPP) to give 4-hydroxyphenyl acetic acid (4-HPA).

(a) Overlay of ^1H NMR spectra (partial spectra are shown for clarity) of a freshly prepared mixture of L-Asc/4-hydroxyphenyl pyruvate (4-HPP)/Tris-D_{11} buffer (bottom) compared with the same mixture after 10 hours (top). (b) Stability of 4-HPP in buffer: overlay of ^1H NMR spectra (partial spectra are shown for clarity) of a freshly prepared mixture of 4-HPP in aqueous Tris-D_{11} buffer (bottom) compared with the same mixture after 10 hours (top). (c) H_2O_2-mediated conversion of 4-HPP to 4-HPA: overlay of ^1H NMR spectra (partial spectra shown for clarity) of a freshly prepared mixture of 4-HPP in aqueous Tris-D_{11} buffer (bottom) compared with the same mixture with H_2O_2 after 25 minutes (top). Concentrations used: 500 µM 4-HPP, 500 µM H_2O_2 in 50 mM aqueous Tris-D_{11} buffer at pH 7.5.

(a)

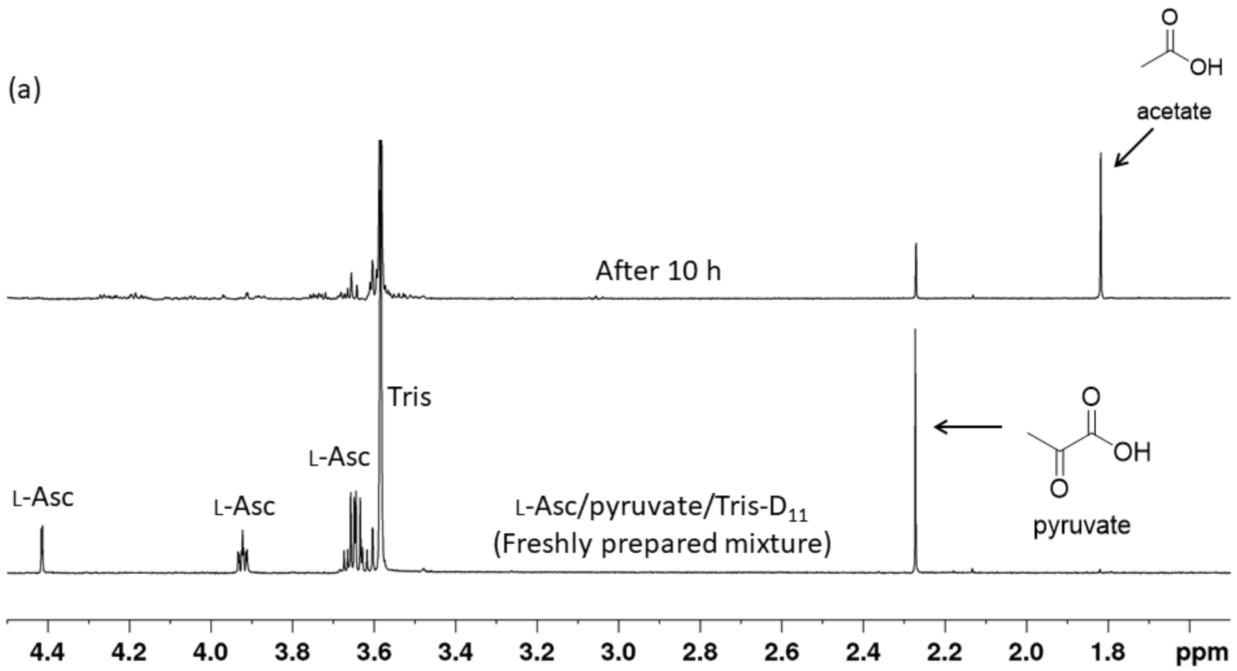

(b)

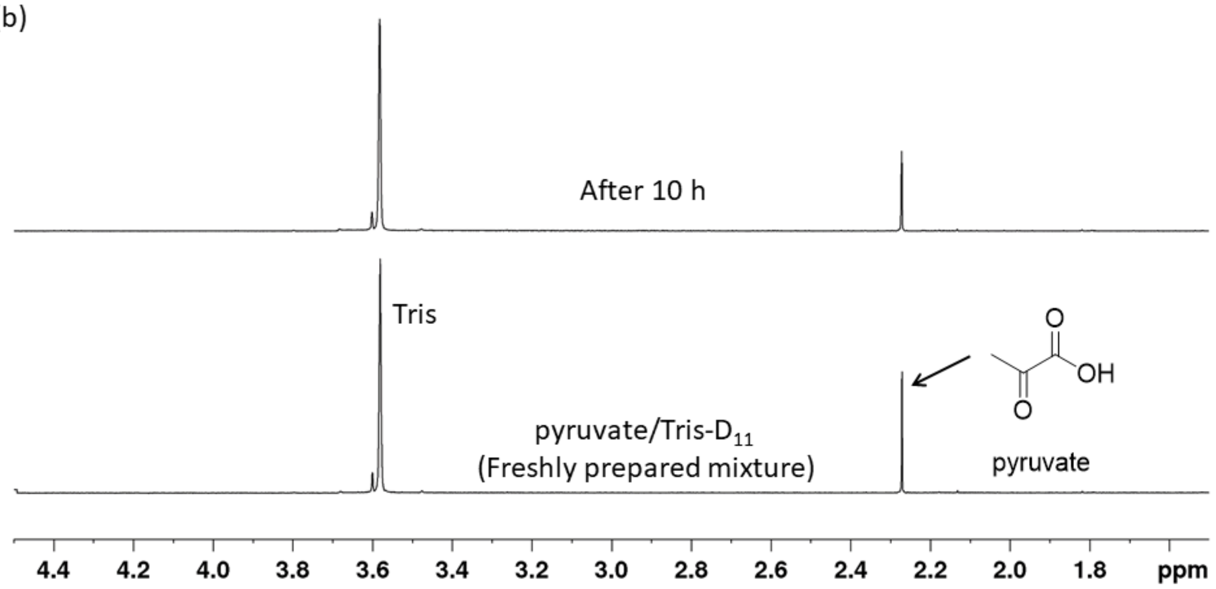

(Figure continues)

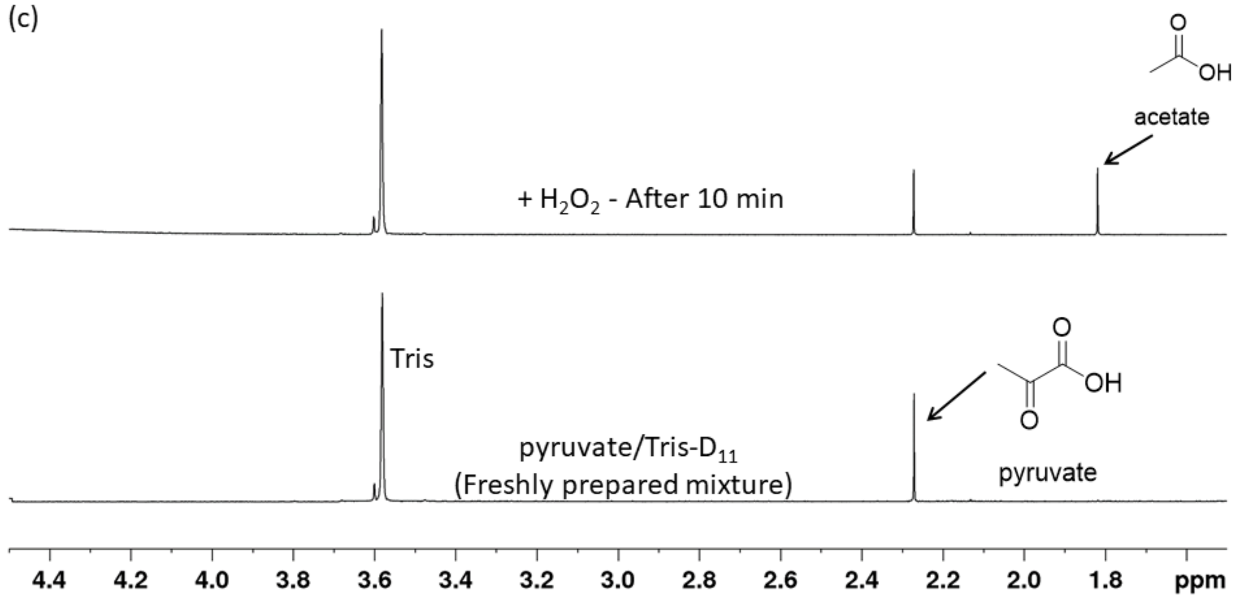

Figure S12. L-Asc-mediated conversion of pyruvate to acetate.
(a) Overlay of ^{1}H NMR spectra of a freshly prepared mixture of L-Asc/pyruvate/Tris-D_{11} buffer (bottom) compared with the same mixture after 10 hours (top). (b) Stability of pyruvate in buffer: overlay of ^{1}H NMR spectra of a freshly prepared mixture of pyruvate in aqueous Tris-D_{11} buffer (bottom) compared with the same mixture after 10 hours (top). (c) H_2O_2-mediated oxidative decarboxylation of pyruvate to give acetate: overlay of ^{1}H NMR spectra of a freshly prepared mixture of pyruvate in aqueous Tris-D_{11} buffer (bottom) compared with the same mixture with H_2O_2 after 10 minutes (top). Concentrations used: 500 µM L-Asc, 200 µM pyruvate, 500 µM H_2O_2 in 50 mM aqueous Tris-D_{11} buffer at pH 7.5.

(a)

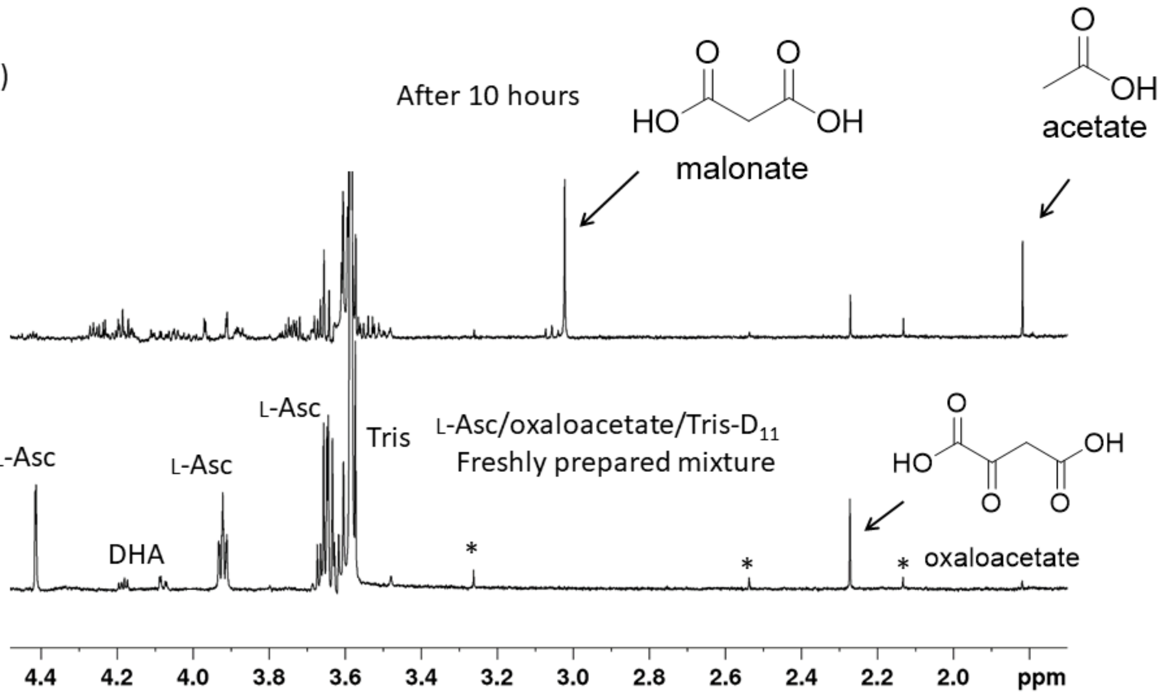

(b)

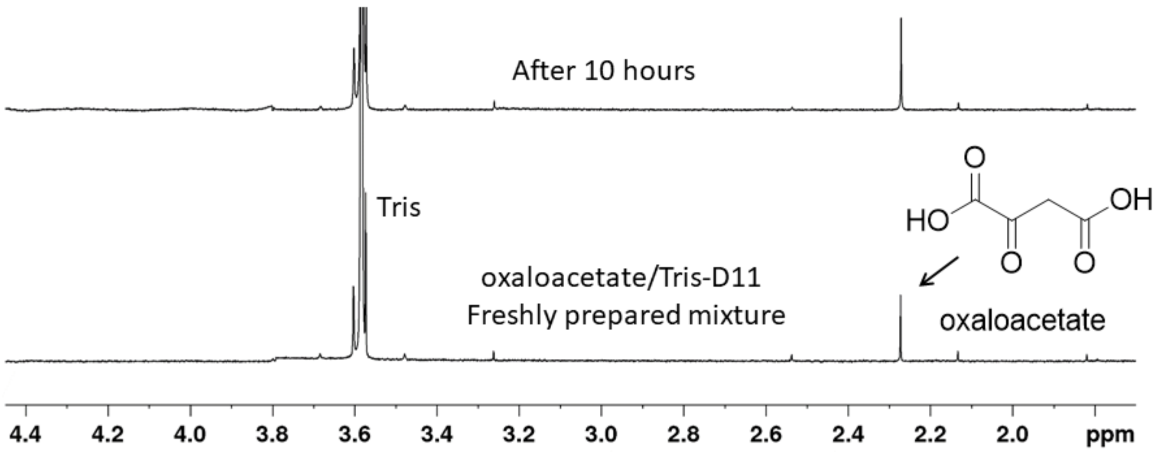

(Figure continues)

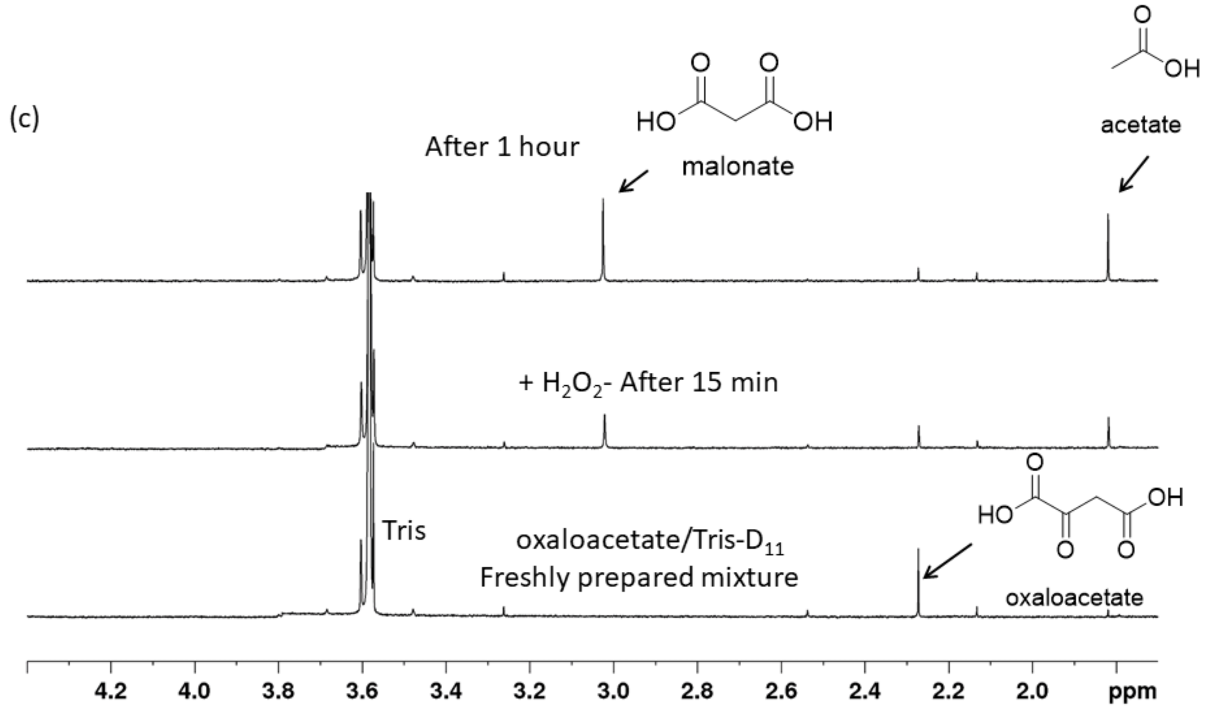

Figure S13. L-Asc mediated conversion of oxaloacetate to malonate and acetate.
(a) Overlay of ^{1}H NMR spectra of a freshly prepared mixture of L-Asc/oxaloacetate/Tris-D_{11} buffer (bottom) compared with the same mixture after 10 hours (top). Peaks marked with asterisks are likely due to impurities. The formation of acetate can occur by non-oxidative decarboxylation of malonate. Note that whereas malonate undergoes facile non-oxidative decarboxylation, succinate does not undergo decarboxylation to give propionate because of the lack of the `β-carbonyl group´ to promote decarboxylation. (b) Stability of oxaloacetate in buffer: overlay of ^{1}H NMR spectra of a freshly prepared mixture of oxaloacetate in aqueous Tris-D_{11} buffer (bottom) compared with the same mixture after 10 hours (top). (c) H_2O_2-mediated conversion of oxaloacetate to malonate and acetate: overlay of ^{1}H NMR spectra of a freshly prepared mixture of oxaloacetate in aqueous Tris-D_{11} buffer (bottom) compared with the same mixture containing H_2O_2 after 15 minutes (middle) and 1 hour (top). Concentrations used: 500 µM L-Asc, 200 µM oxaloacetate, 500 µM H_2O_2 in 50 mM aqueous Tris-D_{11} buffer at pH 7.5.

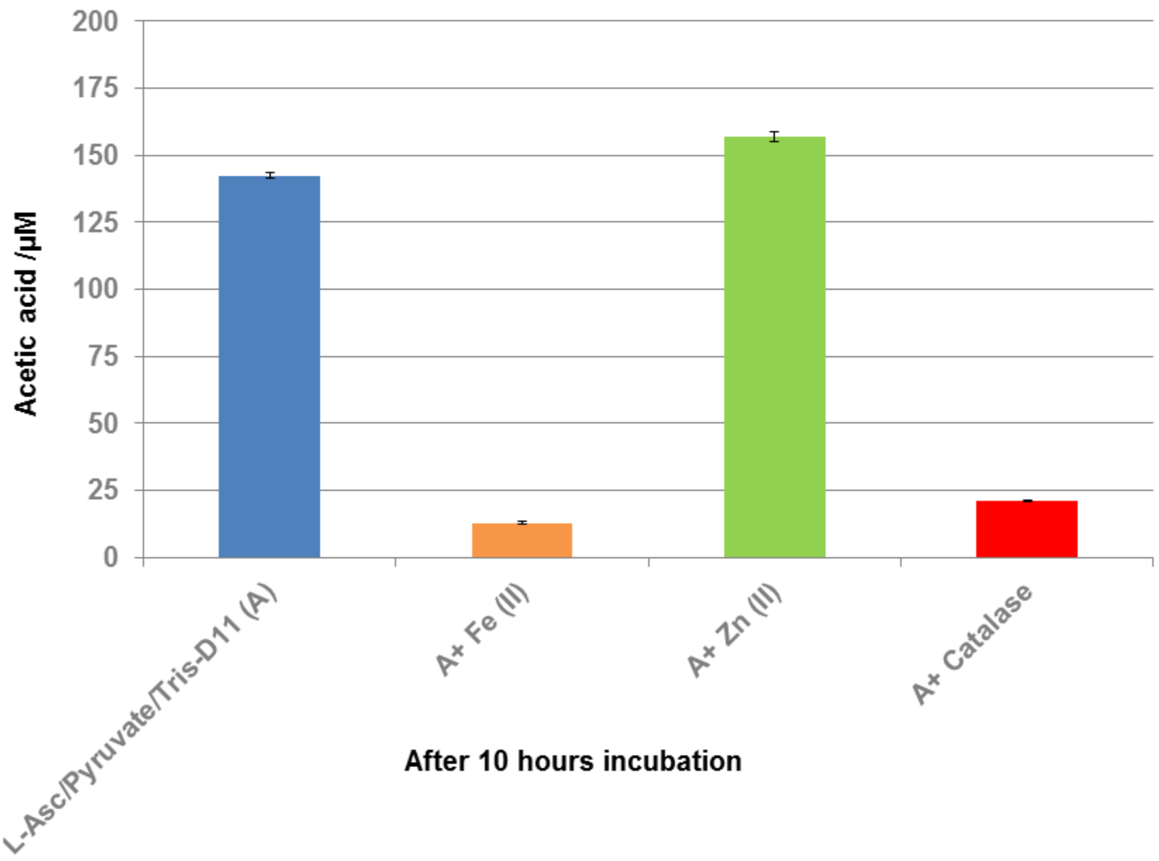

Figure S14. The effects of Fe(II), Zn(II) and catalase on the L-Asc mediated conversion of pyruvate to acetate.

Chart showing the extent of acetate formation after incubation (10 hours) of a mixture of L-Asc/pyruvate/Tris-D_{11} buffer (blue); mixture of L-Asc/pyruvate/Fe(II)/Tris-D_{11} buffer (orange); mixture of L-Asc/pyruvate/Zn(II)/Tris-D_{11} buffer (green); mixture of L-Asc/pyruvate/catalase/Tris-D_{11} buffer (red). Concentrations used: 500 μM L-Asc, 200 μM pyruvate, 100 μM Fe(II), 100 μM Zn(II) and 1735 units catalase in 50 mM aqueous Tris-D_{11} buffer at pH 7.5. These observations show that the addition of either Fe(II) or catalase inhibits the L-Asc mediated reaction of pyruvate to acetate. Error bars represent standard deviations from the mean (n = 3) of three separate measurements.

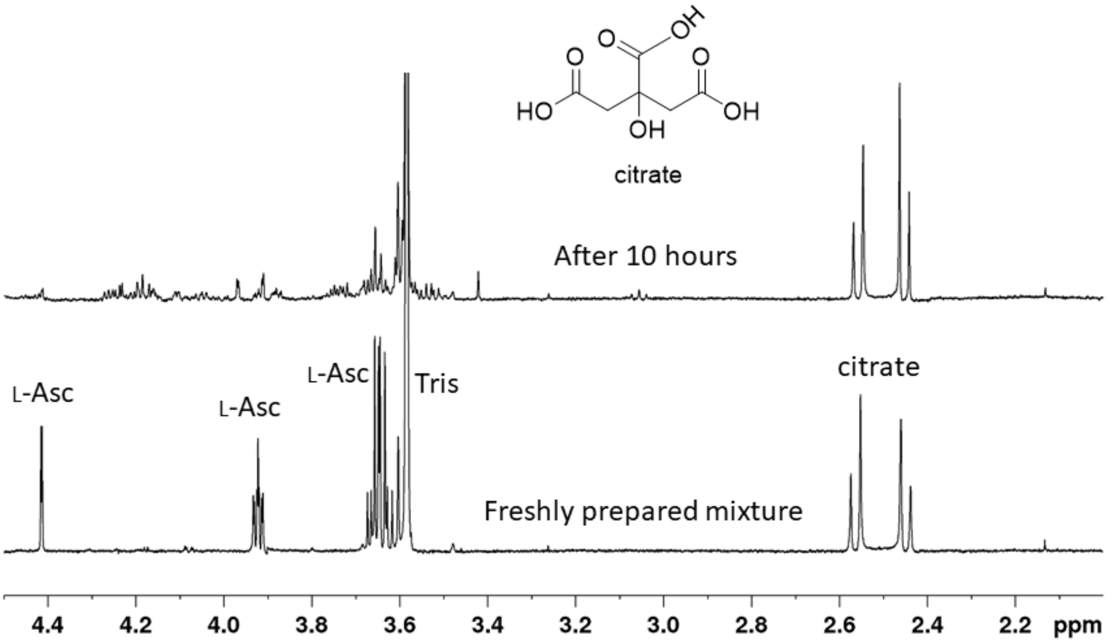

Figure S15. L-Asc and citrate incubation assay.

Overlay of ^{1}H NMR spectra of a freshly prepared mixture of citric acid/L-Asc/Tris-D_{11} buffer compared with the same mixture after 10 hours. No new signals except those assigned to the reaction of L-Asc appear in the spectrum (i.e there is no evidence for citrate reaction). Concentrations used: 500 µM L-Asc, 200 µM citric acid in 50 mM aqueous Tris-D_{11} buffer at pH 7.5.

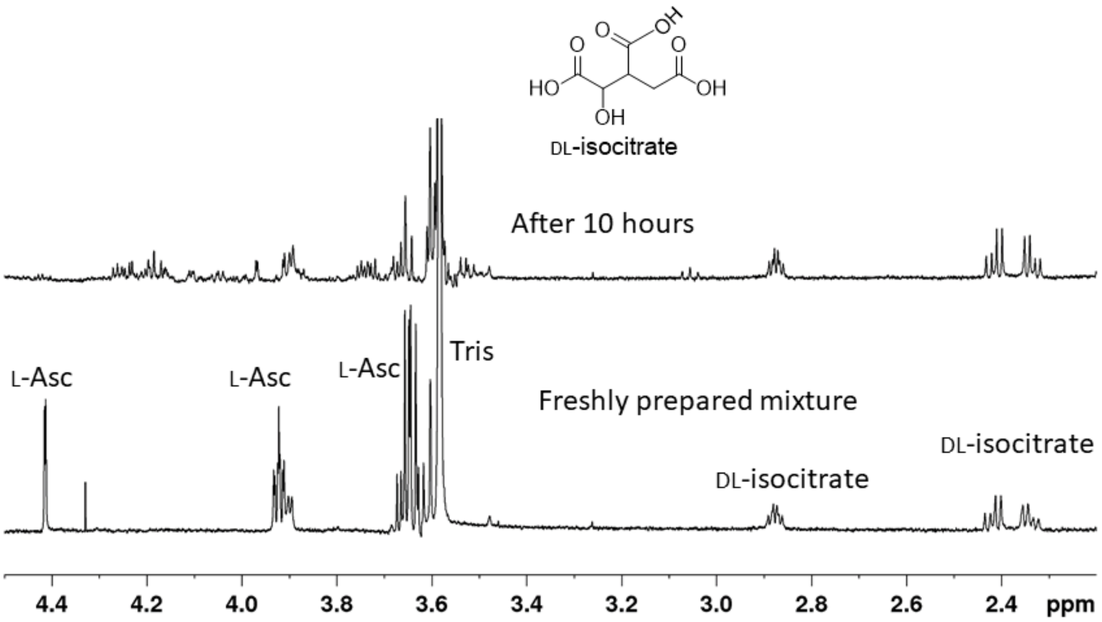

Figure S16. L-Asc and DL-isocitrate incubation assay.

Overlay of ^{1}H NMR spectra of a freshly prepared mixture of DL-isocitrate/L-Asc/Tris-D_{11} buffer compared with the same mixture after 10 hours. No new signals except those assigned to the reaction of L-Asc appear in the spectrum (i.e there is no evidence for the reaction of DL-isocitrate). Concentrations used: 500 µM L-Asc, 200 µM DL-isocitrate in 50 mM aqueous Tris-D_{11} buffer at pH 7.5.

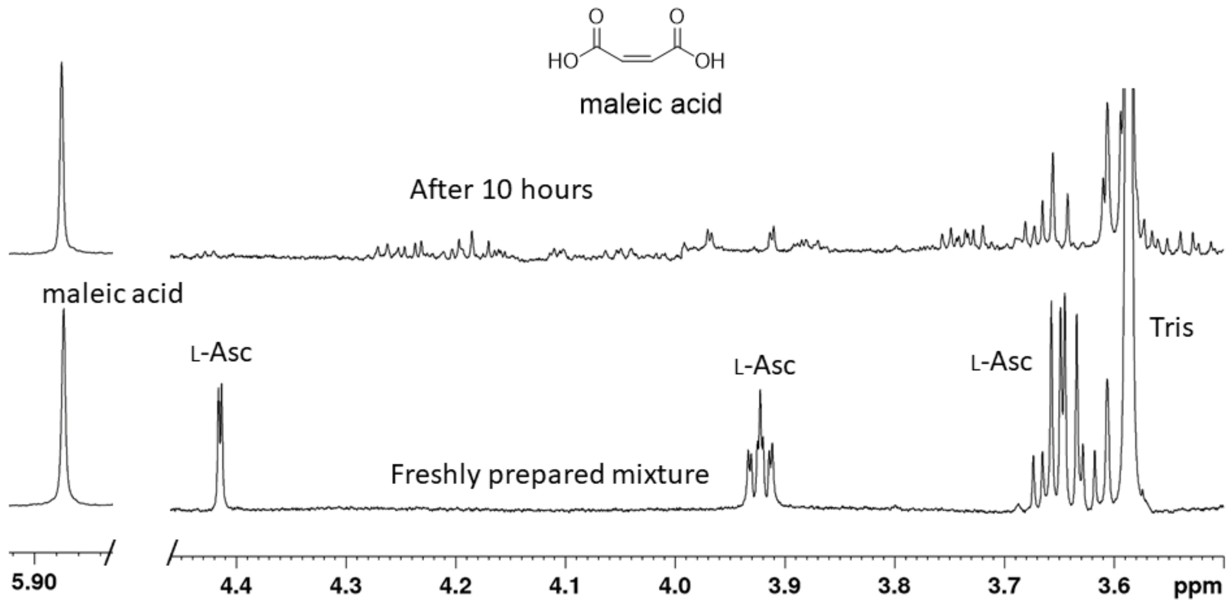

Figure S17. L-Asc and malate incubation assay.

Overlay of ^{1}H NMR spectra (parts are shown for clarity) of a freshly prepared mixture of maleic acid/L-Asc/Tris-D_{11} buffer compared with the same mixture after 10 hours. No new signals except those assigned to the reaction of L-Asc appear in the spectrum (i.e there is no evidence for the reaction of maleic acid). Concentrations used: 500 µM L-Asc, 200 µM maleic acid in 50 mM aqueous Tris-D_{11} buffer at pH 7.5.

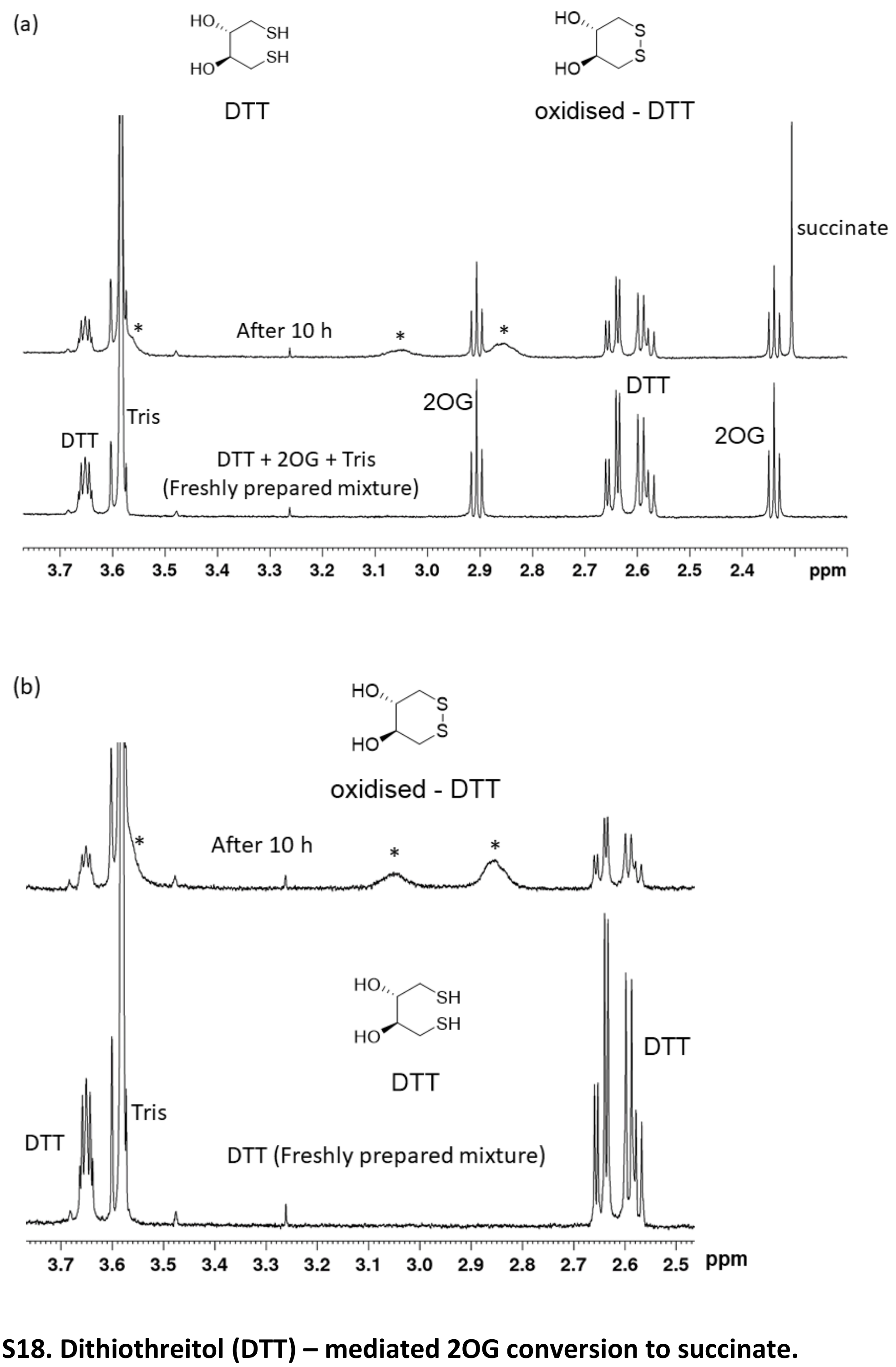

Figure S18. Dithiothreitol (DTT) – mediated 2OG conversion to succinate.

(a) Overlay of ^{1}H NMR spectra of a freshly prepared mixture of DTT/2OG/Tris-D_{11} buffer (bottom) compared with the same mixture after 10 hours (top). (b) Oxidation of DTT with dioxygen in buffer: overlay of ^{1}H NMR spectra of a freshly prepared mixture of DTT in Tris-D_{11} buffer (bottom) compared with the same mixture after 10 hours (top). The broad signals marked with asterisks likely represent an oxidised (di)sulfide form of DTT. Concentrations used: 500 µM DTT, 200 µM 2OG in 50 mM aqueous Tris-D_{11}, pH 7.5.

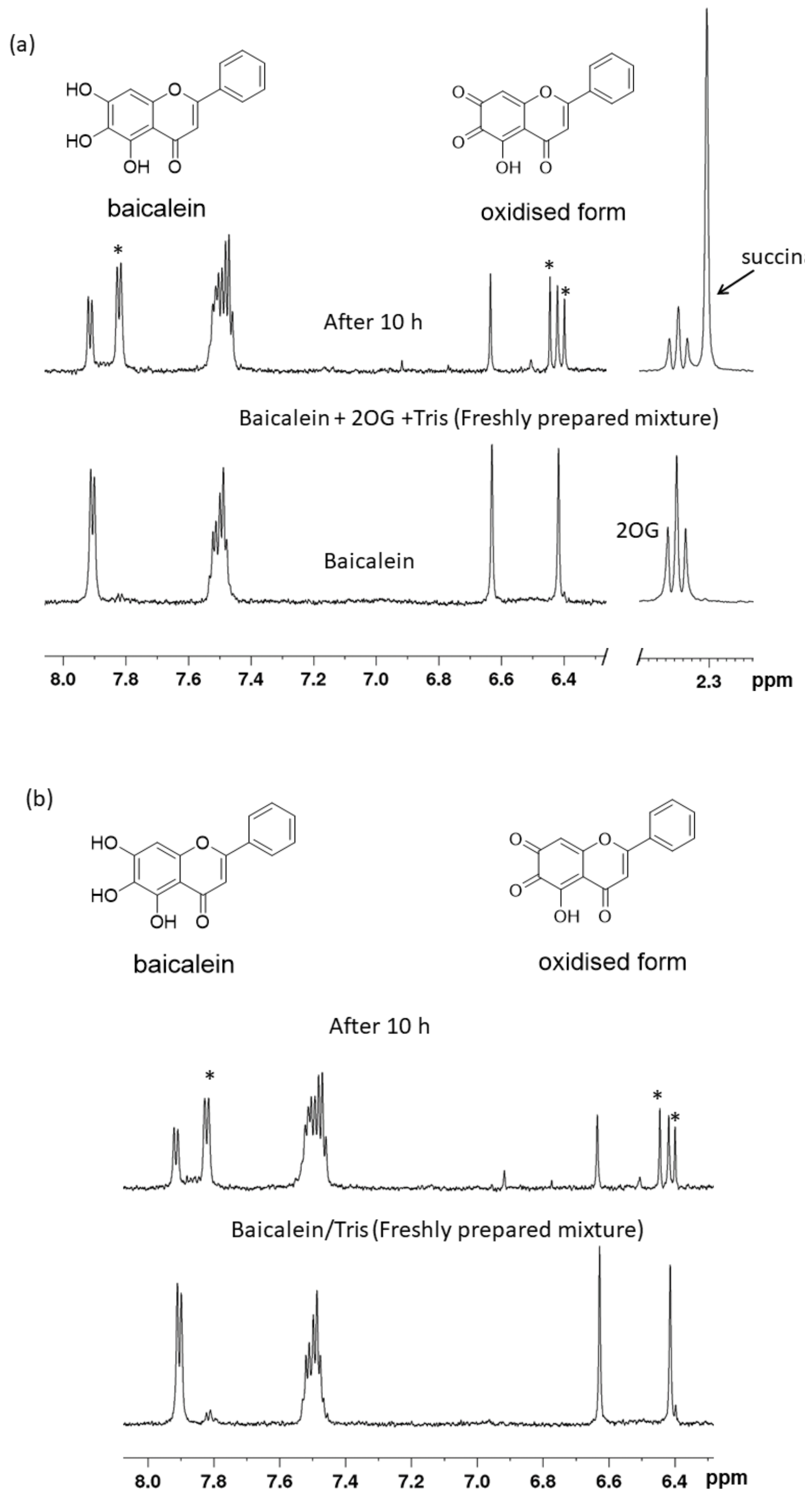

Figure S19. Baicalein-mediated 2OG conversion to succinate.

(a) Overlay of ^{1}H NMR spectra (partial spectra are shown for clarity) of a freshly prepared mixture of baicalein/2OG/Tris-D_{11} buffer (bottom) compared with the same mixture after 10 hours (top). (b) Oxidation of baicalein in buffer: overlay of ^{1}H NMR spectra of a freshly prepared mixture of baicalein in Tris-D_{11} buffer (bottom) compared with the same mixture after 10 hours (top). Signals marked with asterisks are believed to represent an oxidised form of baicalein. Concentrations used: 500 µM baicalein, 200 µM 2OG in 50 mM aqueous Tris-D_{11} buffer at pH 7.5.

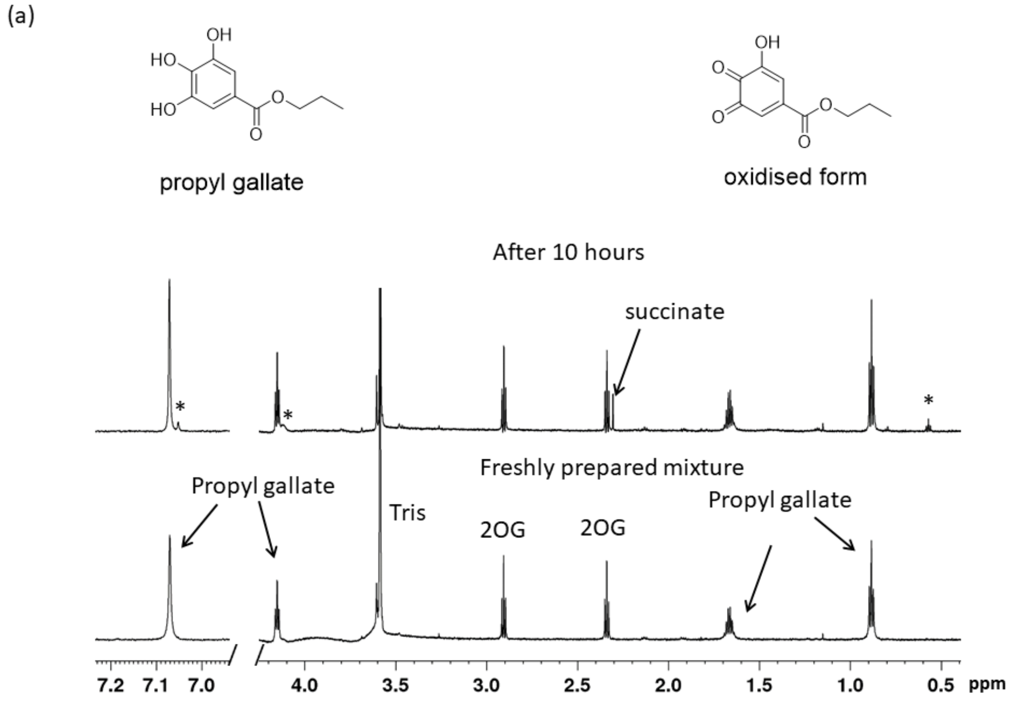

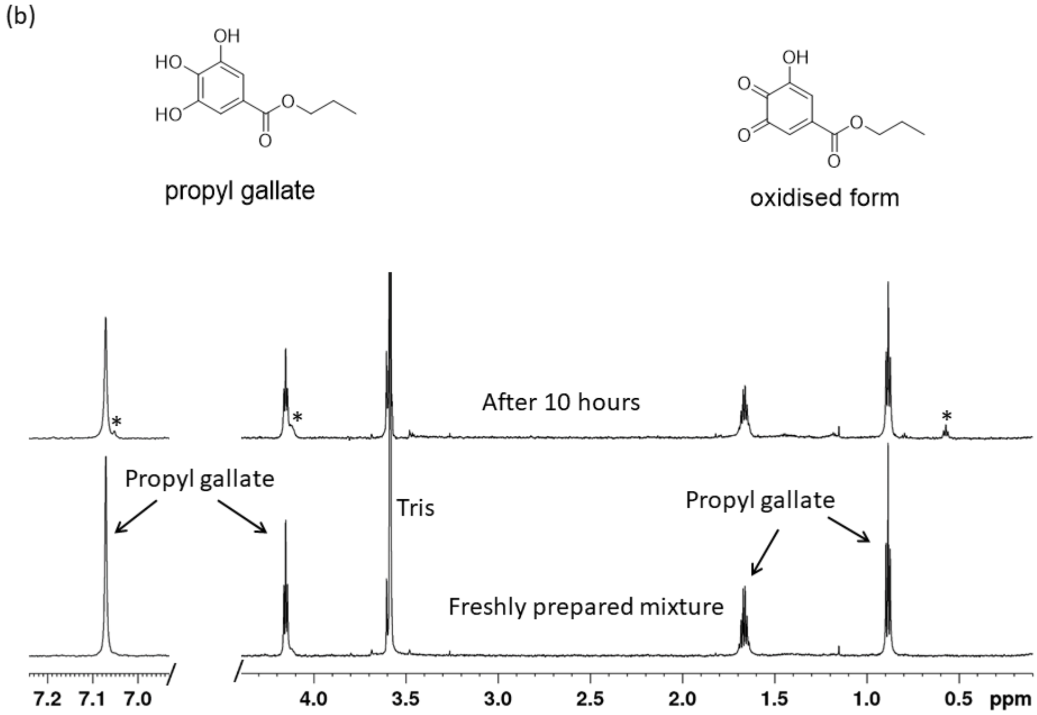

Figure S20. Propyl gallate-mediated conversion of 2OG to succinate.

(a) Overlay of ^{1}H NMR spectra (partial spectra are shown for clarity) of a freshly prepared mixture of propyl gallate/2OG/Tris-D_{11} buffer (bottom) compared with the same mixture after 10 hours (top). (b) Oxidation of propyl gallate with dioxygen in buffer: overlay of ^{1}H NMR spectra (partial spectra are shown for clarity) of a freshly prepared mixture of propyl gallate in Tris-D_{11} buffer (bottom) compared with the same mixture after 10 hours (top). Signals marked with asterisks are believed to be an oxidised form of propyl gallate. Concentrations used: 500 µM propyl gallate, 200 µM 2OG in 50 mM aqueous Tris-D_{11} buffer at pH 7.5.

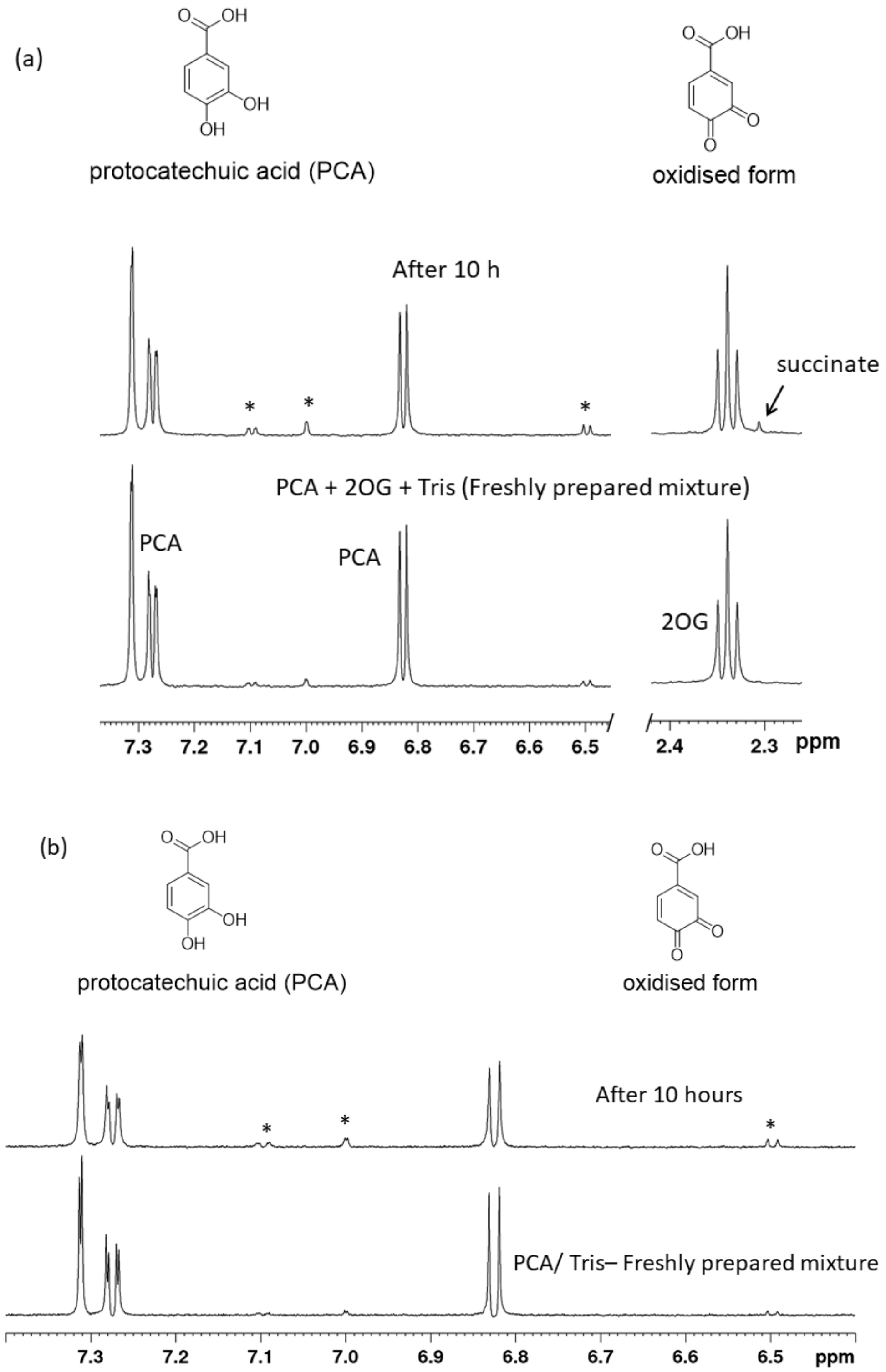

Figure S21. Protocatechuic acid (PCA)-mediated 2OG conversion to succinate.

(a) Overlay of ^{1}H NMR spectra (partial spectra are shown for clarity) of a freshly prepared mixture of protocatechuic acid (PCA)/2OG/Tris-D_{11} buffer (bottom) compared with the same mixture after 10 hours (top). (b) Oxidation of PCA in buffer: overlay of ^{1}H NMR spectra of a freshly prepared mixture of PCA in Tris-D_{11} buffer (bottom) compared with the same mixture after 10 hours (top). Signals marked with asterisks possibly represent an oxidised form of PCA. Concentrations used: 500 µM protocatechuic acid, 200 µM 2OG in 50 mM aqueous Tris-D_{11} buffer at pH 7.5.

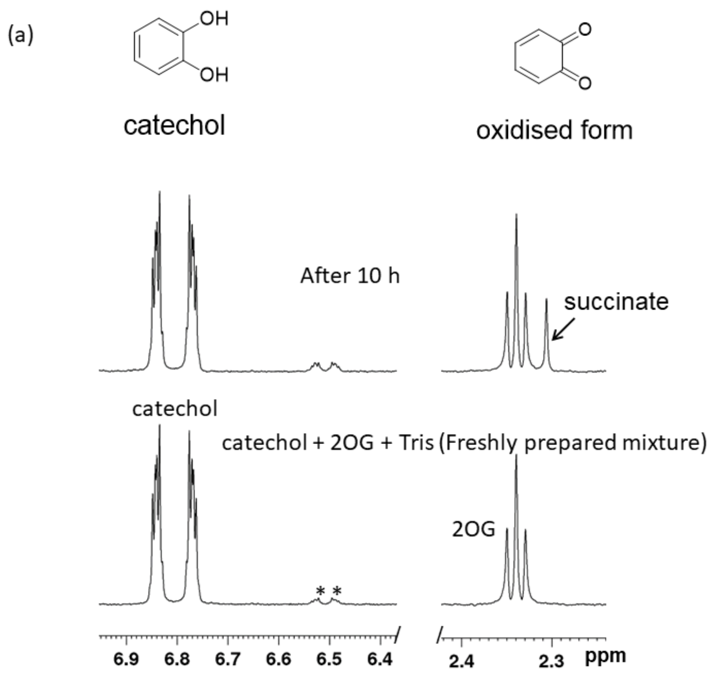

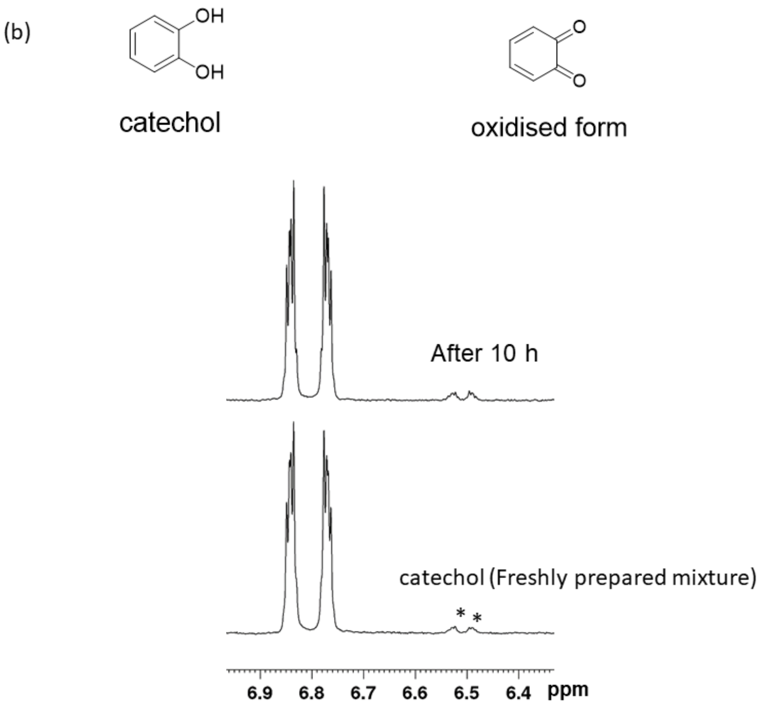

Figure S22. Catechol-mediated 2OG reaction to succinate.

(a) Overlay of ^{1}H NMR spectra (partial spectra are shown for clarity) of a freshly prepared mixture of catechol/2OG/Tris-D_{11} buffer (bottom) compared with the same mixture after 10 hours (top). (b) Oxidation of catechol with dioxygen in buffer: overlay of ^{1}H NMR spectra (partial spectra are shown for clarity) of a freshly prepared mixture of catechol in Tris-D_{11} buffer (bottom) compared with the same mixture after 10 hours (top). Signals marked with asterisks possibly represent an oxidised form of catechol. Concentrations used: 500 µM catechol, 200 µM 2OG in 50 mM aqueous Tris-D_{11} buffer at pH 7.5.

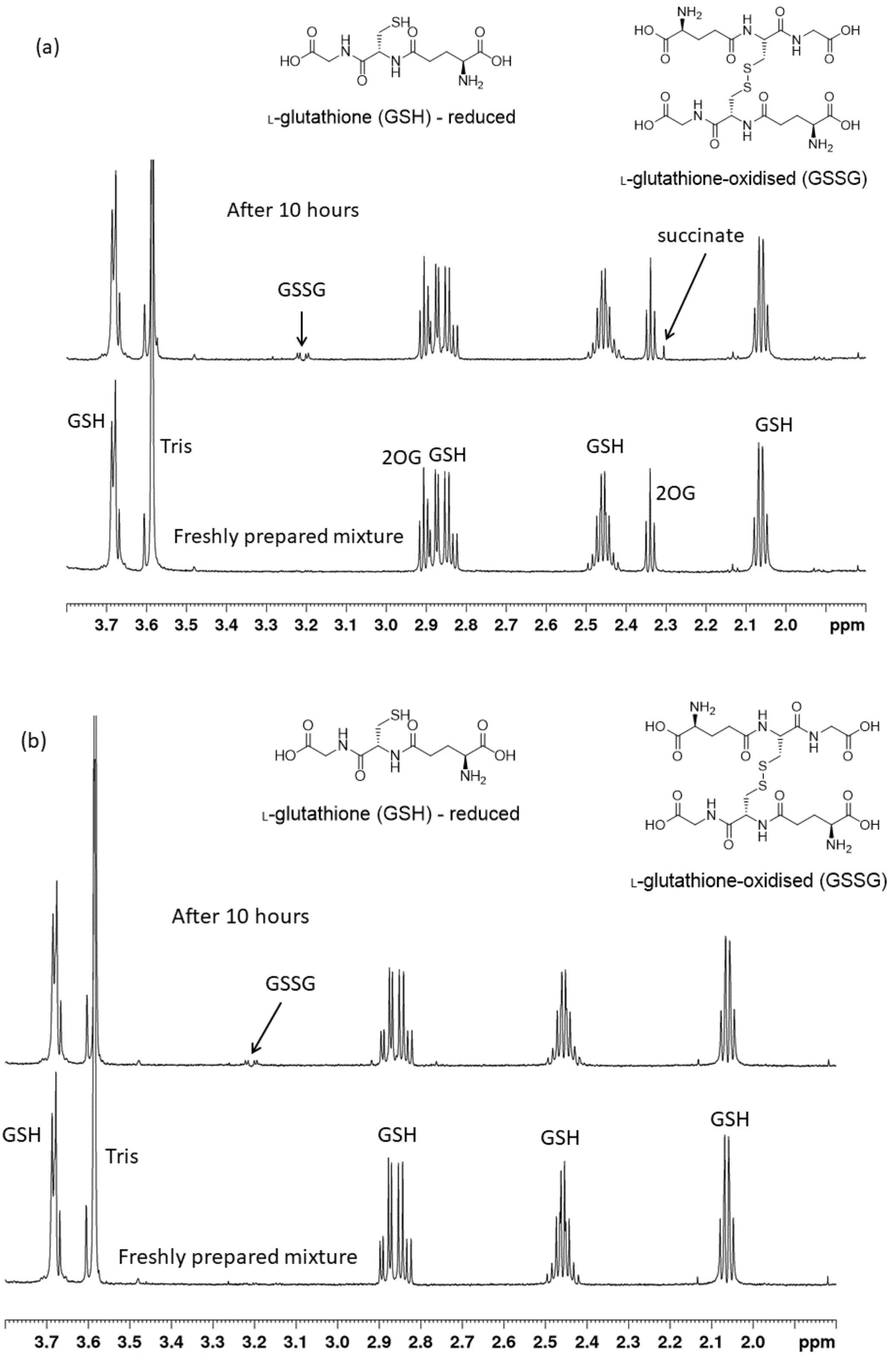

Figure S23. Glutathione (GSH)-mediated 2OG conversion into succinate.

(a) Overlay of ^{1}H NMR spectra of a freshly prepared mixture of glutathione (GSH)/2OG/Tris-D_{11} buffer (bottom) compared with the same mixture after 10 hours (top). (b) Oxidation of GSH in buffer: overlay of ^{1}H NMR spectra of a freshly prepared mixture of GSH in Tris-D_{11} buffer (bottom) compared with the same mixture after 10 hours (top). Concentrations used: 500 µM glutathione, 200 µM 2OG in 50 mM aqueous Tris-D_{11} buffer at pH 7.5. GSSG: Disulfide-glutathione.

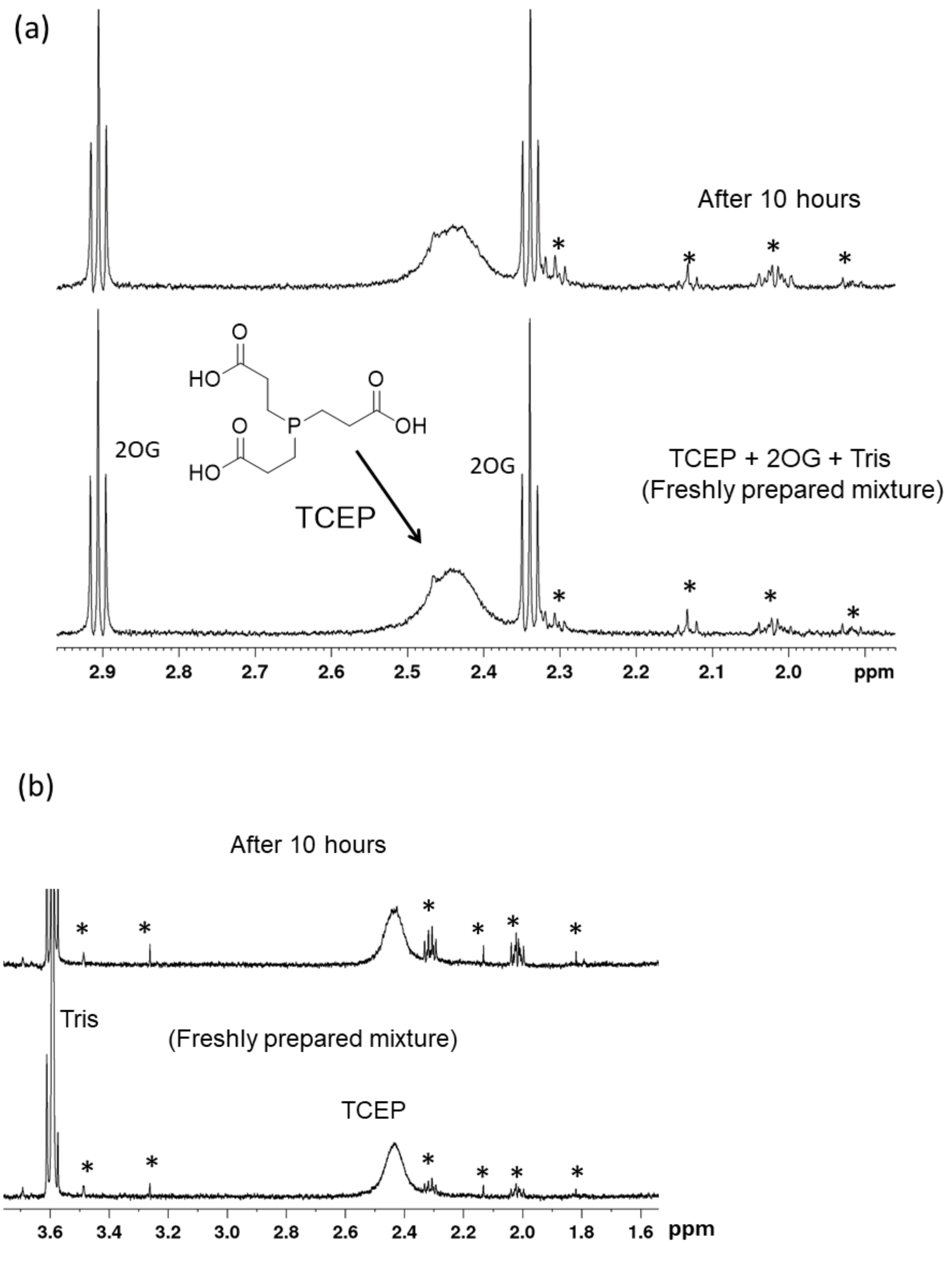

Figure S24. Tris(2-carboxyethyl)phosphine (TCEP) – 2OG incubation assay.

(a) Overlay of ^{1}H NMR spectra (partial spectra are shown for clarity) of a freshly prepared mixture of TCEP/2OG/Tris-D_{11} buffer (bottom) compared with the same mixture after 10 hours (top). (b) TCEP stability in buffer: overlay of ^{1}H NMR spectra of a freshly prepared mixture of TCEP in Tris-D_{11} buffer (bottom) compared with the same mixture after 10 hours (top). The peaks labelled with asterisks may be due to impurities in the TCEP/buffer solution. Concentrations used: 500 µM TCEP, 200 µM 2OG in 50 mM aqueous Tris-D_{11} buffer at pH 7.5.
